# Efficacy of aerosol therapy of lung cancer correlates with EGFR paralysis induced by AvidinOX-anchored biotinylated Cetuximab

**DOI:** 10.18632/oncotarget.2409

**Published:** 2014-08-31

**Authors:** Rita De Santis, Antonio Rosi, Anna Maria Anastasi, Caterina Chiapparino, Claudio Albertoni, Barbara Leoni, Angela Pelliccia, Daniela Santapaola, Valeria Carollo, Emanuele Marra, Luigi Aurisicchio, Brunilde Arseni, Maria Lucrezia Pacello, Gabriella Palmieri, Simone Battella, Fiorella Petronzelli, Ferdinando Maria Milazzo

**Affiliations:** ^1^ Sigma-Tau SpA R&D, Pomezia, Rome, Italy; ^2^ Takis Srl, Via di Castel Romano, Rome, Italy; ^3^ University La Sapienza, Experimental Medicine Department, Viale Regina Elena, Rome, Italy

**Keywords:** AvidinOX, Cetuximab, lung cancer, aerosol

## Abstract

Lung cancer, as well as lung metastases from distal primary tumors, could benefit from aerosol treatment. Unfortunately, because of lung physiology, clearance of nebulized drugs is fast, paralleled by unwanted systemic exposure. Here we report that nebulized AvidinOX can act as an artificial receptor for biotinylated drugs. In nude and SCID mice with advanced human KRAS-mutated A549 metastatic lung cancer, pre-nebulization with AvidinOX enables biotinylated Cetuximab to control tumor growth at a dose lower than 1/25,000 the intravenous effective dose. This result correlates with a striking, specific and unpredictable effect of AvidinOX-anchored biotinylated Cetuximab, as well as Panitumumab, observed on a panel of tumor cell lines, leading to inhibition of dimerization and signalling, blockade of endocytosis, induction of massive lysosomal degradation and abrogation of nuclear translocation of EGFR. Excellent tolerability, together with availability of pharmaceutical-grade AvidinOX and antibodies, will allow rapid clinical translation of the proposed therapy.

## INTRODUCTION

Lung cancer is the leading cause of cancer-related deaths and it has the greatest overall economic burden among all cancers [1]. Many patients are diagnosed with locally advanced disease and receive platinum-based therapies whose efficacy and tolerability are not satisfactory. Lung cancer cells express ErbB receptors and anti-epidermal growth factor receptor (EGFR) monoclonal antibodies (Mab) such as Cetuximab, Panitumumab and Necitumumab have been widely used in clinical trials by intravenous administration, showing limited efficacy and poor tolerability [2-4].

Aerosol may be an appealing delivery route for lung cancer therapy because of site specificity, low drug doses and excellent patient's compliance. Several pre-clinical and clinical studies with nebulized chemotherapeutics [5], gene therapy [6] and Cetuximab [7] showed, in principle, feasibility and utility of such approach. Unfortunately, lung's physiology is well adapted to clear inhaled exogenous substances. Therefore, there is a need to identify new technologies improving lung residency of nebulized therapeutics.

We recently reported that injected AvidinOX exhibits the distinctive property to form Schiff's bases with tissue proteins thus constituting a stable receptor for biotinylated therapeutics [8-12]. This product is currently investigated in phase I clinical trial for targeting ^177^Lutetium-biotinDOTA (^177^Lu-ST2210) [13] to inoperable liver metastases (ClinicalTrials.gov NCT02053324). Presently, we show that aerosol treatment with AvidinOX, followed by a very low dose of biotinylated Cetuximab (bCet) is effective in inhibiting tumor growth, in severe metastatic lung cancer models. This result correlates with an unpredictable improvement of *in vitro* anti-tumor activity of Cetuximab as well as Panitumumab when, in their biotinylated version, the antibodies are anchored to AvidinOX on the surface of tumor cells. Importantly, good tolerability and availability of pharmaceutical-grade AvidinOX and anti-EGFR monoclonal antibodies will allow rapid translation of the proposed treatment in clinical trials.

## RESULTS

**Figure 1:**
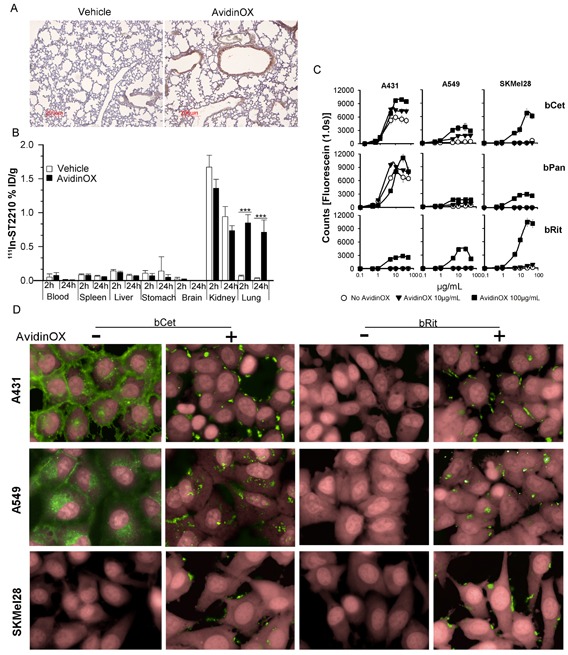
Nebulized AvidinOX sticks to the lung and uptakes intravenous radioactive biotin, and tumor cell-bound AvidinOX prevents biotinylated Cetuximab internalization A, Representative image of Avidin immunostaining of lung sections from vehicle- or AvidinOX-nebulized mice (*n* = 3). AvidinOX (3 mg/mL). B, Biodistribution of ^111^In-ST2210 intravenously injected in mice, 24 h after AvidinOX (3 mg/mL) or vehicle aerosol treatment. Mice were sacrificed 2 or 24 h after radioactive biotin injection. Data are expressed as % injected dose/g (%ID/g) of tissue. Error bars: mean ± s.d. (*n* = 5). Student's *t* test: ***, *p*< 0.001. C, Binding of CF488-labelled bCet, bPan or bRit in the concentration range 0.1-50 μg/mL to the indicated cells pre-conjugated with 10 or 100 μg/mL AvidinOX. Data are mean fluorescence counts of triplicate wells ± s.d. X-axis: log_10_ antibody concentration. D, Cells, without or with AvidinOX conjugation (100 μg/mL) incubated 30 min with 5 μg/mL CF488-labelled biotinylated antibodies (green). Draq5 dye staining of nucleus and cytoplasm (pink). Fluorescence imaging by High Content Screening (HCS) Operetta. Each image is representative of at least 5 fields of triplicate wells. Magnification 60X.

Nebulized drugs are rapidly eliminated from the lung by mechanisms leading to degradation and/or transportation into the blood stream. Immunoglobulins, including Cetuximab, are translocated into the blood by neonatal FcR (FcRn)-mediated transcytosis [14, 15]. We hypothesized that entrapment of anti-EGFR Mabs within the lung might be useful for treating tumors nesting in the lung and we thought to deliver by aerosol biotinylated Cetuximab (bCet) after AvidinOX. Linkage of nebulized AvidinOX to the lung needed to be demonstrated having previously employed it by intra-tissue injection, only. Therefore, we exposed mice to nebulized AvidinOX and found, after 24 h, avidin immunostaining up to terminal bronchiole (Fig. 1A). An AvidinOX dose-escalating study demonstrated uptake of intravenous radioactive biotin (^111^In-ST2210) in the lung, reaching plateau after 40 minute exposure (Supplementary Table S1A). Subsequently, we confirmed that mice, nebulized 40 minutes with AvidinOX, exhibit specific uptake of intravenous ^111^In-ST2210 in the lung and that radioactivity persists at least 24 hours (Fig. 1B). Representative μPET image of mice nebulized with AvidinOX showing distribution of intravenously injected ^64^Cu-ST2210 in the whole lungs in Supplementary Figure S1. Overall data indicate that nebulized AvidinOX links to the lung and it can be used for delivering biotinylated drugs. Radionuclide therapy of lung cancer is deemed impracticable because of the high sensitivity of normal lung to irradiation. Therefore, we decided to investigate the use of AvidinOX for targeting biotinylated Cetuximab, relaying on higher toxicity of the antibody towards tumor compared to normal cells.

To test the effect of AvidinOX anchorage on Cetuximab activity, the antibody was biotinylated according to previous methods [16]. Panitumumab (human IgG2 anti-EGFR) and Rituximab (chimeric IgG1 anti-CD20 Mab) were also biotinylated representing a second EGFR-specific and a negative control Mab, respectively. Similarity of biotinylated Mabs with their original version was confirmed and purity and potency specifications were set to maximize consistency among batches (Supplementary Table S1B). *In vitro* binding and anti-tumor activity of free and AvidinOX-anchored biotinylated antibodies were evaluated on a panel of tumor cell lines of different origin and exhibiting different EGFR expression (high A431, medium H1299, low A549 or none SKMel28) and oncogenic pathways. Tumor cell characteristics in Supplementary Table S1C. AvidinOX conjugation to tumor cells, performed as previously described [17], did not affect the binding properties of Cetuximab (Supplementary Fig. S2A) or Panitumumab (data not shown), as measured by cytofluorimetry. Binding of bCet and biotinylated Panitumumab (bPan) to tumor cells, correlated with the number of cell surface EGFR molecules and biotinylated Rituximab (bRit) did not bind. All biotinylated antibodies bound AvidinOX-conjugated cells independently on the presence of their specific antigen, as expected. Binding of bCet and bPan to EGFR expressing cells appeared to be slightly increased on AvidinOX-conjugated cells compared to unconjugated, possibly as a result of antigen and AvidinOX binding (Supplementary Fig. S2B). Quantitative evaluation of bCet and bPan binding to A431, A549 and SKMel28 cells, pre-conjugated with 10 or 100 μg/mL AvidinOX, confirmed previous cytofluorimetry data and pointed out a pro-zone effect at antibody concentrations higher than 25 μg/mL on cells conjugated with the higher AvidinOX concentration. This effect is independent on antibody specificity (bRit) or antigen expression (SKMel28) thus likely attributable to a competitive binding of biotinylated antibodies to AvidinOX (Fig. 1C). The fate of AvidinOX-anchored antibodies was investigated by High Content Screening (HCS) fluorescence imaging. Fluorescent bCet and bPan but not fluorescent bRit were found within the cytoplasm of A431 and A549 but not SKMel28 cells after 30 minute incubation, as expected. On AvidinOX-conjugated cells, fluorescence was observed on the membrane of all cells and interestingly, in this condition, internalization of biotinylated anti-EGFR antibodies was prevented (Fig. 1D). Internalization of EGFR/ligand (EGF or anti-EGFR antibodies) complex is a physiological mechanism affecting the tumor cell response to growth and inhibition stimuli. We then addressed the effect of Cetuximab anchorage on tumor cell proliferation.

**Figure 2:**
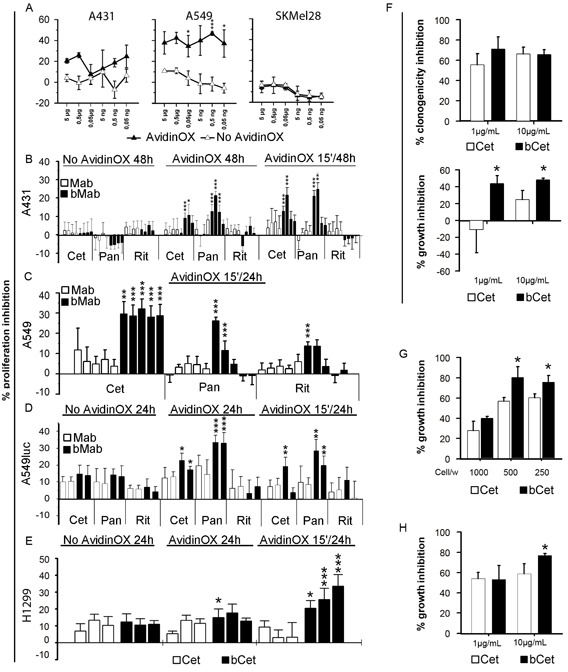
Biotinylated Cetuximab and Panitumumab inhibit growth of AvidinOX-conjugated tumor cells A, Cells cultivated in 96 well plates 48 h after initial 15 min contact with bCet at the indicated concentrations. Cell viability measured by CellTiter-Glo assay. Data are expressed as % inhibition versus control (medium). Error bars: mean of 6 wells ± s.d. Student's *t* test AvidinOX versus No AvidinOX: ***, p<0.001; *, p<0.05. B, Cells cultivated 48 h with indicated antibodies at 10-2-0.4-0.08 μg/mL. 15′/48h indicates antibody removal after initial 15 min contact. XTT added the last 3 h. Data expressed as % inhibition versus control. Replicates and error bars as in A. C, Cells cultivated 24 h, after initial 15 min contact, with indicated antibodies at 200-20-2-0.2-0.02 μg/mL. 5BrdU added the last 4 h. Replicates and error bars as in A. D, Cells cultivated 24 h with indicated antibodies at 20 or 2 μg/mL. 15′/24h indicates antibody removal after initial 15 min contact. Light emission measured by Bioluminescence Assay (Caliper). Replicates and error bars as in A. E, Cells cultivated 24 h with Cet or bCet at 0.2, 2 and 20 μg/mL. 15′/24h indicates cultures where antibodies were removed after initial 15 min contact. 5BrdU added the last 4 h. Data are expressed as % inhibition versus control. Replicates and error bars as in A. F, A549 cells seeded in 24 well plates (100 cells/well) and, after 2 h adherence, bCet and Cet in DMEM 1% FBS, added with and without AvidinOX conjugation, respectively. After 14 days, cultures were fixed, stained with crystal violet and clones counted (upper panel). Growth inhibition (size of clones), measured as OD_595_ values of stained clones eluted with 30% acetic acid (lower panel). Data are expressed as % inhibition versus control. Error bars: mean of six wells ± s.d. G, A549 cells seeded in triplicate, in 6 well plates at the indicated number/well. 10 μg/mL bCet or Cet, in DMEM 1% FBS, added with and without AvidinOX conjugation, respectively. After 7 days, the cultures of 1000 cells/well were fixed and stained with crystal violet. In the cultures of 500 and 250 cells/well, the medium was replaced without antibodies for additional 7 day cultivation. At the end, cultures were fixed, stained and clones eluted as in F. Data are expressed as % inhibition versus control. H, A431 cells tested as in F. Panels B-H, Student's *t* test bMab versus Mab: ***, *p*<0.001; **, *p*<0.01; *, *p*<0.05. In all experiments, AvidinOX conjugation was performed at 100 μg/mL. All panels: representative data from at least two independent experiments.

**Figure 3:**
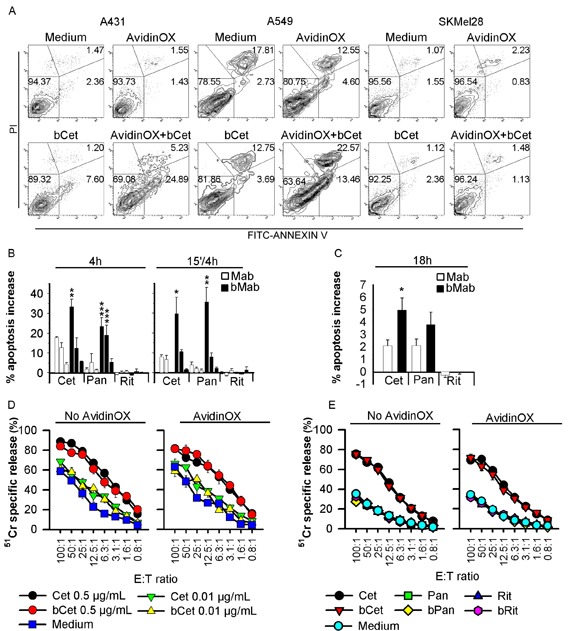
Biotinylated Cetuximab and Panitumumab induce apoptosis of AvidinOX-conjugated cells, and AvidinOX anchorage does not affect Cetuximab-mediated ADCC A, Cytofluorimetry of Annexin V^+^ cells without (medium) or with AvidinOX conjugation, following 15 min incubation with 5 μg/mL bCet and 18 h cultivation. B, A431 cells cultivated in triplicate with indicated antibodies at 5-0.5-0.05 μg/mL, 4 h or the first 15 min only. Annexin V^+^ cells detected by cytofluorimetry. Data expressed as % increase of Annexin V^+^ cells versus control. Error bars: mean ± s.d. Student's *t*-test bMab versus Mab: ***, *p*<0.001; **, *p*<0.01; *, *p*<0.05. C, A549 cells cultivated 18 h in triplicate with 5 μg/mL of indicated antibodies, and Annexin V^+^ cells detected as in B. Data are mean ± s.e. of three independent experiments. Anova, bMab versus Mab: *, *p*<0.05. D, NK-mediated ADCC. Target: ^51^Cr-labelled A549 cells incubated 20 min with Cet or bCet at indicated concentrations Effector: human PBMC-derived primary NK cells. Cultures were typically > 85% NK (CD56^+^CD16^+^CD3^−^), as assessed by flow cytometry. Data are mean of triplicate wells ± s.e. E, Same as in D with indicated antibodies at 0.5 μg/mL. In all experiments, AvidinOX conjugation was performed at 100 μg/mL. All panels: representative data from at least two independent experiments.

It was previously demonstrated that proliferation of PC3 (prostate carcinoma), 3T3 and mouse spleen cells is not affected by AvidinOX conjugation [17]. This result was also preliminarily confirmed with A431, A549, H1299 and SKMel28 cells (data not shown). On the other hand, biological activity of AvidinOX-anchored bCet and bPan needed to be confirmed. Unexpectedly, higher proliferation inhibition of A431, A549 and H1299 (EGFR^+^) but not SKMel28 (EGFR^−^) cells was induced by AvidinOX-anchored bCet or bPan compared to Cet or Pan, even after only 15 minute contact, as to simulate a transient exposure to drugs like in aerosol treatment (Fig. 2A-E). Interestingly, lower inhibitory activity was occasionally observed at the highest antibody concentration reminding of the pro-zone effect previously seen in binding experiments (Fig. 1C). It is to note that inhibition of A549 by Cetuximab and Panitumumab was only obtained with concentrations higher than 200 μg/mL where Rituximab also occasionally inhibited (data not shown). On the contrary, on AvidinOX-conjugated cells, bCet and bPan were active at doses below 2 μg/mL. Clonogenic assay showed significant difference in the size but not number of A549 or A431 cell clones with AvidinOX-anchored bCet compared to Cet (Fig. 2F-H). Inhibition of EGFR^+^ tumor cell growth by AvidinOX-anchored anti-EGFR antibodies correlated with increased induction of apoptosis (Fig. 3A-C). Proliferation and apoptosis experiments were performed with 10 and 100 μg/mL AvidinOX conjugation and most reproducible data, here shown, were obtained with the higher AvidinOX concentration.

It is known that antibody-dependent cell cytotoxicity (ADCC) contributes to the overall clinical efficacy of Cetuximab [18]. Therefore, we deemed necessary to test the effect of AvidinOX anchorage on Cetuximab-mediated ADCC. Results showed that ADCC activity of human primary NK cells against A549, is comparable if mediated by Cet or AvidinOX-anchored bCet (Fig. 3D). Rituximab, Panitumumab and their biotinylated derivatives were negative in all conditions (Fig. 3E). These data indicate that AvidinOX anchorage does not impair Cetuximab ADCC and it does not activate antigen-(Rituximab) or isotype-(Panitumumab) independent ADCC.

**Figure 4:**
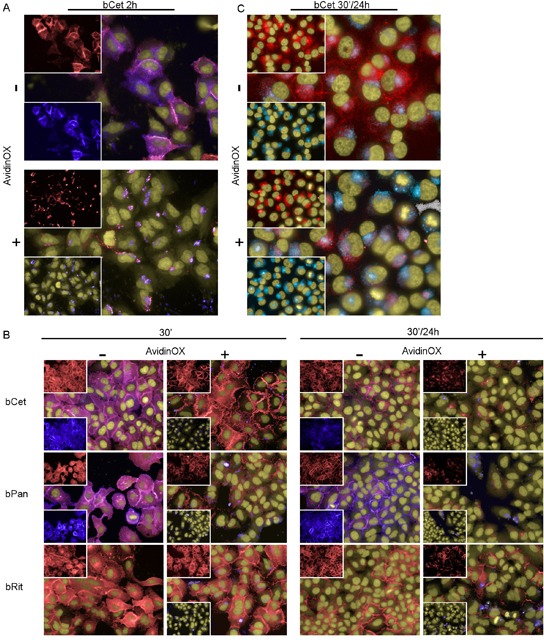
AvidinOX-anchored bCet and bPan inhibit endocytosis and induce degradation of EGFR Fluorescence imaging by High Content Screening (HCS) Operetta of A549 cells, with or without AvidinOX conjugation (100 μg/mL), incubated with 5 μg/mL CF488-labelled bMabs (blue). At indicated time, cells were washed, fixed and stained for the detection of EGFR by AF555-labeled anti-EGFR Mab (D38B1) (red). Draq5 dye staining of nucleus and cytoplasm (yellow). Violet is the result of blue and red dye co-localization in the merged images. A, bCet, 2 h cultivation. B, bCet, bPan or bRit, 30 min or 30 min contact and 24 h cultivation. C, Unlabelled bCet 30 min contact and 24 h cultivation. Staining of lysosomes by LysoTracker (light blue) and staining of EGFR as before. Hoechst dye staining of nuclei (yellow). All panels: representative picture of at least 5 fields of triplicate wells. Magnification 60X.

EGFR family uses a distinctive mechanism of receptor homo-hetero-dimerization [19] followed by endocytosis of EGFR/ligand (or antibody) complex and signaling [20, 21]. Our data indicate that bMab internalization is prevented by AvidinOX (Fig. 1D). Interference of AvidinOX conjugation with EGFR endocytosis was preliminarily ruled out in EGF-stimulated A431, A549 and H1299 cells by fluorescence imaging (Supplementary Fig. S3A). The possibility that AvidinOX-mediated entrapment of anti-EGFR bMabs on the cell membrane, might interfere with EGFR trafficking was then addressed. Without AvidinOX, EGFR rapidly co-localized with bCet within the cytoplasm of A549 cells while with AvidinOX, bCet remained co-localized with EGFR on the cell membrane and the intracellular EGFR appeared to be significantly reduced after 2 hours (Fig. 4A). This effect was proved specific as it was not observed with AvidinOX-anchored bRit (Supplementary Fig. S3B) while it was similar with bPan on A431, A549 and H1299 cells (Supplementary Fig. S3C). Reduction of intracellular EGFR in A549 cells was evident after 30 minute antibody contact and almost complete at 24 hours (Fig. 4B). Similar results were obtained with H1299 cells (Supplementary Fig. S3D). Disappearance of intracellular EGFR in A549 cells in the presence of AvidinOX-anchored bCet correlated with co-localization of EGFR within lysosomes indicating massive receptor degradation (Fig. 4C). A large size representative image of EGFR and lysosome co-localization in Supplementary Figure S3E. bPan but not bRit induced the same co-localization effect (Supplementary Fig. S3F). Fluorescence imaging data indicate that AvidinOX anchorage of anti-EGFR antibodies prevents their internalization as well as endocytosis of EGFR. This sort of “receptor paralysis” is associated to massive translocation of intracellular EGFR in the lysosomes resulting in massive degradation. To investigate this peculiar phenomenon at molecular level, we performed western blot experiments.

**Figure 5:**
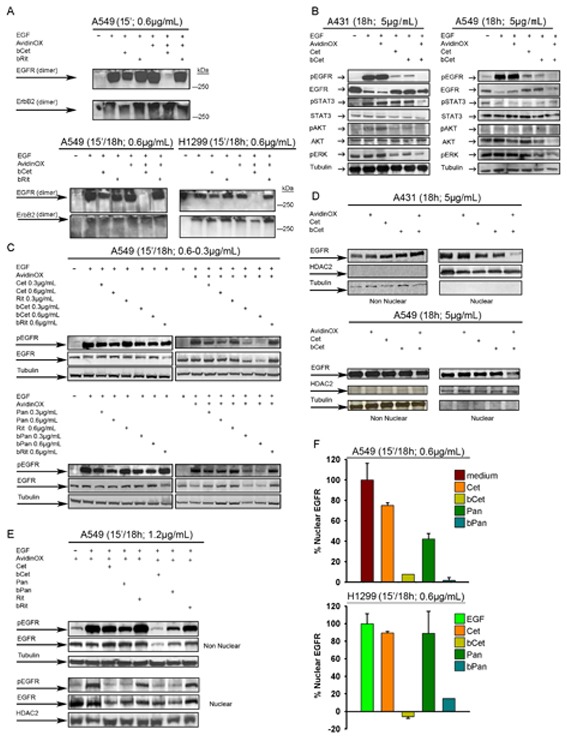
AvidinOX-anchored bCet and bPan inhibit dimerization and signalling of EGFR Cells were serum-starved 24 h and then cultivated, with or without AvidinOX conjugation (100 μg/mL) at indicated conditions. Whole cell lysates or sub-cellular fractions were subjected to Western blot analysis for indicated target proteins. A, Whole cell lysates stimulated 30 min with 1μg/mL EGF and cross-linked with 40mM glutaraldehyde before electrophoretic separation. B, Whole cell lysate. C, Whole cell lysate. D, Nuclear and non-nuclear fractions of not starved cells. E, Nuclear and non-nuclear fractions. F, ELISA titration of nuclear EGFR (PathScan; Cell Signaling). Data are expressed as % residual nuclear EGFR. Error bars: mean ± s.d. (*n* = 3). EGF (100 ng/mL) was added 30 min before cell lysis, where indicated. All panels: representative data from at least two independent experiments.

It is known that EGFR dimers initiate downstream signaling [22]. Interestingly, we found that EGFR homo and heterodimerization but not ErbB2 homodimerization were specifically inhibited by AvidinOX-anchored bCet (Fig. 5A). EGF-induced phosphorylation of EGFR, STAT3, AKT and ERK in A431 and A549 cells was not affected by AvidinOX conjugation and it was similarly inhibited by Cet and bCet in the absence of AvidinOX while, in AvidinOX-conjugated cells, phosphorylation of EGFR was abrogated by bCet (Fig. 5B). Upon this condition, phosphorylation of downstream signalling elements was also strongly reduced, with the exception of pSTAT3 in A549 cells, consistently with the presence of constitutively activated JAK2-mediated STAT3 pathway [23]. Dramatic inhibition of EGFR phosphorylation was observed in AvidinOX-conjugated A549 (Fig. 5C) and H1299 cells (Supplementary Fig. S4) upon 15 minute contact with bCet or bPan but not bRit paralleled by an equally dramatic decrease in the level of total EGFR. A novel nuclear mode of EGFR signalling has been recently described in which EGFR is shuttled from the cell surface to the nucleus after endocytosis and there it acts as a transcriptional regulator inducing proliferation, DNA repair, replication and chemo-, radio-resistance [24, 25]. Cet and bCet caused a similar slight reduction of nuclear EGFR in A431 but not A549 cells while, in both AvidinOX-conjugated cells, bCet caused almost complete EGFR disappearance (Fig. 5D). Reduction of phosphorylated and total EGFR in both nuclear and non-nuclear compartments of EGF-induced A549 cells, after 15 minute contact with bCet or bPan in Figure 5E. Abrogation of nuclear EGFR by bCet or bPan at a concentration as low as 0.6 μg/mL, was confirmed by ELISA in AvidinOX-conjugated A549 and H1299 cells (Fig. 5F). These data, together with previous fluorescence imaging data (Fig. 4A-C and Supplementary S3B-F), indicate that AvidinOX-anchored anti-EGFR antibodies inhibit dimerization, endocytosis and promote massive degradation of EGFR thus preventing nuclear translocation.

**Figure 6:**
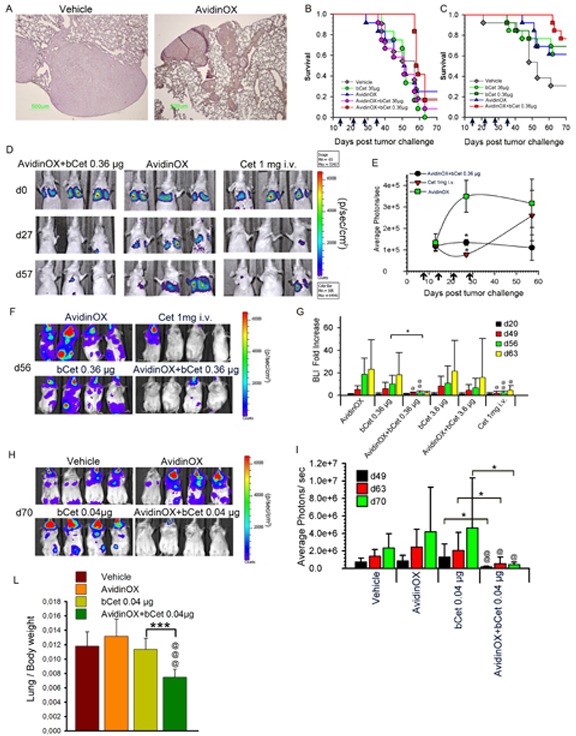
Aerosol treatment with AvidinOX and bCet is effective against A549 metastatic lung cancer A, Representative image of Avidin immunostaining of nude mice lung (*n* = 12), 10 weeks after A549 transplantation (6×10^6^ cells i.v.) and 7 days after the 8^th^ weekly aerosol exposure to nebulized AvidinOX or vehicle. Magnification 4×. B, Kaplan-Meier of nude mice groups (*n* = 12), i.v. transplanted with A549 cells as before. Aerosol, indicated by arrows, started two weeks after tumor challenge. C, Same experimental condition as in B. D, Representative bioluminescence images (BLI) of 3 out of 12 nude mice, i.v. injected with A549luc cells, the day of tumor challenge (d0), one day before 4^th^ weekly nose-only aerosol treatment (d27) and 29 days after the last treatment (d57). E, Average of light photons/sec from lung of study in D. Data are mean ± s.d. Student's *t*-test versus AvidinOX: *, *p*< 0.05. F, Representative bioluminescence images (BLI) of 4 out of 12 SCID/bg mice, i.v. injected with A549luc cells, one week after the 4^th^ weekly nose-only aerosol or i.v. treatment (d56). Treatment started 3 weeks after tumor challenge. G, Lung BLI data of study in F. Fold increase of BLI versus BLI at one day before first treatment (d20). Subsequent measurements were performed one day after 4^th^ treatment (d49), and one and two weeks after last treatment (d56 and 63, respectively). Data are mean ± s.d. Student's *t*-test versus bCet: *, *p*< 0.05. Student's *t*-test versus AvidinOX: @@, *p*< 0.01; @, *p*<0.05. H, Representative bioluminescence images (BLI) of 4 out of 12 SCID/bg mice, i.v. injected with A549luc cells, one week after 8^th^ weekly, whole-body aerosol treatment (d70). Treatment started one week after tumor challenge. I, Average photons of study in H. Measurements were performed after 6^th^ and 8^th^ treatment (d49 and 63, respectively) and one week after the last treatment (d70). Data are mean photons ± s.d. Student's *t*-test versus bCet: *, *p*< 0.05. Student's *t*-test versus AvidinOX: @, *p*< 0.05; @@, *p*< 0.01. L, Lung/body weight ratio of study in H, at day 70. Student's *t*-test versus bCet: ***, *p*< 0.001. Student's *t*-test versus AvidinOX:@@@, *p*< 0.001. In all studies, AvidinOX was administered by nebulizing 6.5 mL of 3 mg/mL solution.

*In vitro* data pointed to an unpredictable potentiation of Cetuximab and Panitumumab anti-tumor activity upon AvidinOX anchorage, thus supporting their use, in combination with AvidinOX, for aerosol therapy of tumors nesting in the lung. Integrity of nebulized AvidinOX, bCet and bPan was preliminarily confirmed by chromatography (Supplementary Fig. S5A). In a previous study with Cetuximab-sensitive A431 cells (intra-tracheal transplantation), 2 mg/mouse Cetuximab, sprayed weekly into upper respiratory tract, induced reduction of tumor nodule size [7]. To test our therapy, we established a more challenging model of metastatic lung cancer by intravenously injecting KRAS mutated, Cetuximab-resistant A549 cells. Lung histopathology from A549-transplanted and AvidinOX-nebulized mice, confirmed solid tumor growth and avidin immunostaining (Fig. 6A). In a first study, mice were treated by nose-only aerosol starting two weeks after A549 transplantation, for 4 consecutive weeks, with an estimated lung delivered dose of 36 μg/mouse bCet , with and without prior 72 μg/mouse AvidinOX nebulization. One group received 0.36 μg/mouse bCet after AvidinOX. Details on aerosol equipment and dose calculation [26] in Supplementary Figure S5B. Unexpectedly, a delay of 28 days on the onset of mortality was observed in the group treated with AvidinOX and 0.36 μg/mouse bCet compared to all other groups (Fig. 6B). A second study confirmed that 0.36 μg bCet was only effective in mice pre-treated with AvidinOX (Fig. 6C). In a third study, bioluminescence imaging (BLI) of nude mice transplanted with A549luc cells showed that mice treated by intravenous Cetuximab (1 mg/mouse) or by aerosol with AvidinOX and bCet (0.36 μg/mouse) exhibited significant reduced light signal in the lung compared to AvidinOX-treated mice (Fig. 6D). After discontinuation of treatment, light emission increased in mice treated with nebulized AvidinOX or with intravenous Cetuximab while the signal remained low in mice treated with nebulized AvidinOX and bCet up to day 57 (Fig. 6E). A fourth study with A549luc cells was performed in SCID/beige mice that are defective of NK activity thus being possibly cured by anti-EGFR inhibition only. Differently from nude mice, BLI pictures of SCID mice showed extensive whole body tumor growth with stronger light signals in head than in lung. Systemic tumor burden was significantly reduced by the treatment with intravenous Cetuximab and, unexpectedly, by aerosol with AvidinOX and 0.36 μg/mouse bCet (Fig. 6F). Quantitative evaluation of the lung's light emission confirmed significant lower signal in mice treated by 4 weekly intravenous injections of 1 mg Cetuximab and in those treated by aerosol with AvidinOX and 0.36 but not 3.6 μg/mouse bCet, compared to controls (Fig. 6G). An additional study was performed in SCID mice by using, for aerosol treatment, a standard household nebulization equipment and delivering, with and without previous AvidinOX, 3.5 mL bCet at 100, 30, 10 and 3 μg/mL, corresponding to 0.4, 0.12, 0,04, 0.012 μg/mouse, respectively. Details on aerosol equipment and dose calculation in Supplementary Figure S5C. BLI, one day after 8 weekly aerosol treatments, strikingly confirmed elimination of whole body light signals in mice treated with AvidinOX followed by a dose as low as 0.04 μg/mouse bCet (Fig. 6H). Average photon data indicated significant anti-tumor efficacy of the treatment during the study and persisting up to one week after treatment discontinuation (Fig. 6I). Similar anti-tumor efficacy was observed with 0.12 μg/mouse while 0.012 and 0.4 μg/mouse showed lower or none activity (Supplementary Fig. S5D). Consistently with BLI data, the ratio of lung/body weight was significantly lower in the group treated with 0.04 μg/mouse (Fig. 6L) in agreement with the effect of treatment on lung tumor burden. Finally, confirmation of the strikingly anti-tumor efficacy of the aerosol treatment with AvidinOX and bCet 0.04 μg/mouse, was obtained by histology showing in this group of mice, one week after the last treatment, an almost normal lung tissue (Supplementary Fig. S5E).

**Figure 7:**
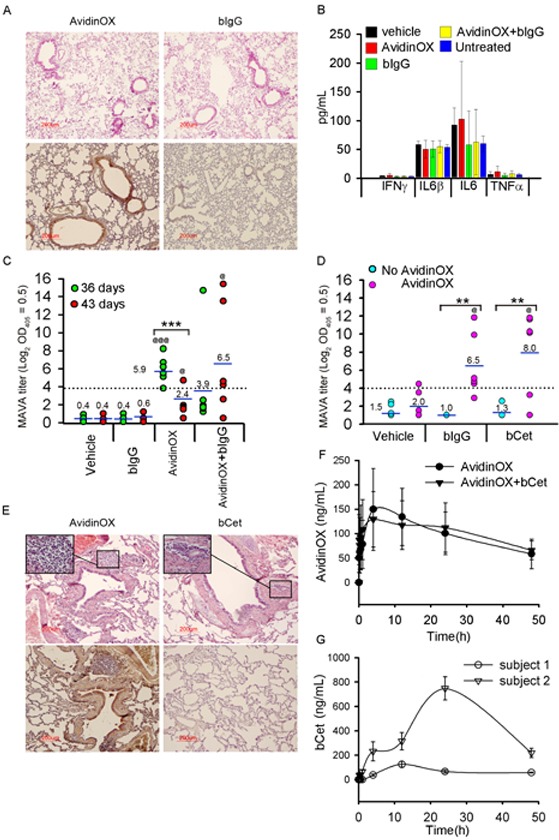
Tolerability to nebulized AvidinOX and/or biotinylated antibody, and AvidinOX immunogenicity A, Balb/c mice nebulized (nose-only) once/week for 6 weeks with AvidinOX (6.5 mL of 3 mg/mL solution), with and without subsequent bIgG (3.5 mL of 30 μg/mL solution), and sacrificed 1 or 7 days after last administration. Representative images (*n* = 6) of lung H/E (upper) and avidin immunostaining (lower), 1 day after the last administration of indicated compounds. B, ELISA titration of inflammatory cytokines in bronchoalveolar lavage of mice as in A. Error bars: mean ± s.d. C, Individual MAVA titers of mice as in A expressed as Log_2_ serum dilution giving OD_405_= 0.5. Mean values indicated by number. Student's *t* test: ***, *p*<0.001; Student's *t* test versus bIgG: @@@, *p*<0.001; @, *p*<0.05. D, Individual MAVA titers of mice (*n* = 6) nebulized (whole body) once/week for 6 weeks with AvidinOX (6.5 mL of 3 mg/mL solution), with and without subsequent bIgG or bCet (3.5 mL of 30 μg/mL solution), and sacrificed 7 days after last administration. Data expressed as in C. Student's *t* test: **, *p*<0.01. Student's *t* test versus bIgG or bCet: @, *p*<0.05. E, Representative picture of cynomolgus lung H/E (upper) and avidin immunostaining (lower), 2 days after intra-tracheal nebulization of AvidinOX (3.0 mg/kg of 3 mg/mL solution) or bCet (12.6 mg/animal of 3 mg/mL solution). Inset: inflammatory infiltrates, magnification 40×. F, Blood kinetics of AvidinOX in Cynomolgus treated with nebulized AvidinOX, with and without subsequent nebulized bCet at doses indicated in E. Error bars: mean of triplicates ± s.d. (*n* = 2). G, Blood kinetics of bCet in Cynomolgus treated with nebulized bCet as in F. Error bars: mean of triplicates ± s.d. (*n* = 2). No bCet was detectable in animals pre-nebulized with AvidinOX.

Pre-clinical development of biopharmaceuticals is more challenging for inhaled compared to parenteral forms because it requires studies addressing both local and systemic toxicities. Particularly, being AvidinOX a xenogenic protein, studies in immunocompetent animals were performed to address immune-related risks. Mice, subjected to six weekly nose-only aerosol treatments with AvidinOX, with and without subsequent biotinylated mouse IgG (bIgG), showed neither clinical signs nor body or lung weight changes (data not shown). Consistently, neither inflammatory signs were found by lung histology despite prominent AvidinOX immunostaining (Fig. 7A) nor difference in bronchoalveolar lavage (BAL) inflammatory cytokines were observed (Fig. 7B). Significantly higher mouse anti-AvidinOX antibody (MAVA) titers were detected in mice exposed to AvidinOX compared to vehicle or bIgG (Fig. 7C). A second study was performed by whole body aerosol exposure. Mice were treated once a week for 6 consecutive weeks with nebulized AvidinOX with and without subsequent nebulized bIgG or bCet. Neither clinical signs nor changes in body or lung weight were recorded and lung histology and BAL cytokine analyses confirmed lack of inflammation (data not shown). Consistently with previous nose-only data, higher MAVA titers were found in mice treated with AvidinOX compared to vehicle, bIgG or bCet (Fig. 7D). Interestingly, MAVA titers were significantly higher in mice that had received bIgG or bCet after AvidinOX thus suggesting a possible adjuvant role of immunoglobulins in AvidinOX immunogenicity. Neither anti-IgG nor anti-Cetuximab antibody response was detectable (data not shown). IgA were detectable in serum but not in BAL of two high responder mice. Average MAVA titers, induced by repeated aerosol exposure to AvidinOX, appear to be lower than mouse, rat and cynomolgus titers after single or repeated i.m. or intra-hepatic injections (Supplementary Fig. S5F). Local tolerability and systemic exposure of intra-tracheal nebulized AvidinOX, bCet and AvidinOX followed by bCet, were then evaluated in cynomolgus monkeys (*Macaca fascicularis*), which is a relevant species for Cetuximab specificity [27]. No clinical signs nor changes in behavior were observed in the 48 hours after treatment. Mild inflammatory signs with minor difference among groups were found in the lung by histology (Fig. 7E). AvidinOX showed a blood peak in all monkeys, 4 hours after nebulization (Fig. 7F). bCet peaked after 12 or 24 hours only in animals not previously treated with AvidinOX (Fig. 7G) confirming that systemic exposure to nebulized bCet is prevented by entrapment of biotinylated antibody in lung-bound AvidinOX.

## DISCUSSION

Aerosol treatment of lung cancer has a strong rationale as a standalone therapy for patients with inoperable, locally advanced disease and as adjuvant to chemotherapy for patients with metastases out of the lung. Aerosol is an attractive option because patient's compliance is high, it can be performed in an outpatient setting, dose of drugs is lower compared to systemic delivery with consequent reduction of systemic side effects and costs. Here we show, in mice pre-nebulized with AvidinOX, anti-tumor efficacy of nebulized bCet. The concentration of nebulized bCet solution appears to be critical. In fact, the lung delivered dose of 0.36 μg/mouse, delivered nebulizing 30 μg/mL solution in the first four efficacy studies was consistently active, whereas the doses of 36 and 0.4 μg/mouse, delivered nebulizing 3 mg/mL and 100 μg/mL solution in the first and fifth study, respectively, were not active. The dose of 0.04 μg/mouse, delivered nebulizing 10 μg/mL solution, was also active, representing the lowest effective dose in the present animal model, as the dose of 0.012 μg/mouse (3 μg/mL solution) was not sufficient. Overall *in vivo* anti-tumor results, in agreement with previous *in vitro* data, suggest that a pro-zone effect in AvidinOX binding might occur at high nebulized bCet concentrations and indicate that the effective lung delivered dose of bCet can be as low as 0.04 μg/mouse, when administered after AvidinOX nebulization. This dose is 1/50.000 of Cetuximab nebulized effective dose previously reported [7] and 1/25,000 the effective 1 mg/mouse intravenous dose that, in turn, corresponds to the 250 mg/m^2^ weekly maintenance dose administered to lung cancer patients for 16-18 weeks in clinical trials. In USA, 18 week Cetuximab treatment costs an average of 80,000$ [28] and the clinical trials, in combination with chemotherapy, were rather disappointing thus casting doubts on the appropriateness of further investigation [4, 29]. A phase 2 trial of Panitumumab in combination with chemotherapy did not improve the clinical outcome of lung cancer patients [30]. Necitumumab, added to chemotherapy, was reported to improve of 1.6 month the overall survival of lung cancer patients (SQUIRE study) and FDA approval is pending (Eli Lilly web site). The cost of Necitumumab is expected to be similar to that of Cetuximab consequently, its clinical applicability is expected to be similarly affected by cost/utility evaluations. No doubts there is still a high medical need for more effective and economically affordable lung cancer therapies. Here we show that aerosol treatment with a very low dose bCet, nebulized after AvidinOX, can control tumor growth within and outside the lung. The most plausible explanation of this unexpected result, derives from increasing awareness of the ability of tumor cells to mediate long-range stimulation of metastatic growth, by releasing systemically microvesicles loaded with epigenetic information [31]. Further studies will be performed to address this issue and better evaluate the consequence of lung cancer inhibition by our aerosol approach, on distal metastases.

Immunocompetent animals show excellent tolerability to repeated AvidinOX exposure, despite the induction of an antibody response, that is expected against a xenogenic protein. In this respect, we previously argued that anti-avidin antibodies do not hamper the human clinical use of Avidin or AvidinOX [17, 32].

Present *in vitro* data point to a new, unpredictable effect on tumor cells of AvidinOX-anchored bCet and bPan that, despite engaging two distinct receptor epitopes [33], both prevent homo and heterodimerization and induce massive lysosomal degradation of EGFR. This result is of particular relevance as there is a growing appreciation of the role of spatial regulation of receptors tyrosine kinase (RTKs) in cancer [34]. Several data indicate that the translocation of cell surface receptors of the EGFR family (EGFR, EGFRvIII, ErbB-2, ErbB-3 and ErbB-4) as well as other receptors (i.e. fibroblast growth factor receptor, vascular endothelial growth factor receptor, insulin-like growth factor receptor, cMET), from the cell membrane to the nucleus is involved in stimulation of cell proliferation, tumor progression, DNA repair and chemo-radio-resistance [25, 35, 36]. Cetuximab-induced nuclear localization of EGFR has been reported [20] and poor response to Cetuximab was observed in lung cancer and other solid tumors correlating with EGFR nuclear localization [35, 37, 38] thus making nuclear EGFR an important molecular target in cancer [39]. Consequently, many groups are trying to inhibit EGFR internalization and nuclear localization. It is known that EGFR internalizes via dynamin- and Syntaxin 6- dependent processes [40, 41] and trafficks within vesicles to subcellular compartments including lysosomes where it is degraded, endoplasmic reticulum where it is recycled back to the cell surface, and nucleus where it sustains pro-tumorigenic signalling, through pathways mutual to ErbB2 [42]. A recent work in A431 cells, where EGFR internalization was impaired by inhibiting dynamin with a small molecule or with siRNA, showed reduction of EGF-mediated STAT3 phosphorylation but not of EGFR or Erk1/2 phosphorylation [43]. Another study in dynamin-depleted murine fibroblasts also showed impairment of EGFR endocytosis but, in contrast with A431 results, a strong enhancement of phosphorylation of the membrane-bound EGFR was observed, paired by unaffected MAPK and increased AKT activation [40]. Phosphorylation of ERK was also previously described in HeLa cells in which EGFR endocytosis was impaired by siRNA silencing clathrin [44]. The overall balance of EGFR trafficking towards degradation or signalling very much affects the response of tumor cells to EGFR inhibitors. Here we clearly show that engagement of EGFR by AvidinOX-anchored biotinylated Cetuximab or Panitumumab prevents dimerization, induces inhibition of phosphorylation and promotes massive degradation of the receptor. Our strategy is more effective in silencing EGFR pathway than inhibiting EGFR endocytosis by any means. Overall picture is schematically represented in Supplementary Figure S6.

Our data provide a proof of concept on utility of an innovative therapeutic platform based on nebulized AvidinOX enabling a smarter use of well-known antibodies like Cetuximab. The choice of a full length antibody, instead of an antibody fragment, was deemed necessary to preserve the ADCC property of Cetuximab. Nevertheless, in future studies, we will address the possibility to use biotinylated antibody fragments, like Fabs or single chain Fvs, which might be more convenient than full size immunoglobulins for pharmacokinetics and production costs. Our approach, that proved to be well tolerated, is broadly useful and highly appealing because: first it will make possible to simultaneously block internalization of multiple tumorigenic receptors (i.e. EGFR, ErbB2, ErbB3 and cMet) that are all of therapeutic value in lung cancer [45, 46], thus representing a precious tool to counteract tumor resistance to single agent therapies; second, it will allow the delivery of diverse, multiple, patient-tailored, biotinylated therapeutics, possibly combining anti-tumor drugs with immune-stimulating reagents like IL2 [47] or anti-CTLA4, anti-PD-1 antibodies [48] thus offering a unique opportunity for an integrated fight against lung cancer, with or without concurrent chemotherapy. In this respect, here we show that AvidinOX anchoring does not interfere with Cetuximab capability to induce ADCC; third, much better tolerability is expected compared to intravenous antibody treatments due to low doses and no systemic exposure; fourth, applicability could be easily extended to lung metastases from any cancer for which therapeutic antibodies are available; last but not least, AvidinOX is not expensive and the amount of expensive antibodies is expected to be so low that the therapy will be certainly affordable for any patient. A pre-clinical regulatory program is ongoing for rapidly moving the proposed aerosol therapy into clinical development.

## METHODS

### HCS Fluorescence Imaging

Cells were seeded in 96-well microtiter plates (3-6×10^3^/well) and cultivated 3 days, starved for additional 24 h in serum-free medium and then, with or without AvidinOX conjugation (100 μg/mL), incubated with 5 μg/mL CF488-labelled antibodies in DMEM, for 30 min. Antibodies were removed by washing with medium and cells analysed for fluorescence by High Content Screening (HCS) system Operetta (Perkin Elmer), immediately, or after 24 h cultivation in medium 10% FBS. Detection of lysosomes by LysoTracker (Life Technologies) added the last 30 min of culture. EGFR was detected by AF555-conjugated rabbit anti-EGFR (D38B1) (Cell Signaling), added after cell fixation with 4% paraformaldehyde in PBS, permeabilization with PBS 0.2%Tween-20 (PBS-T) and blocking with 2% BSA in PBS-T. Cells were counterstained with Draq5 or Hoechst33342 dyes (Cell Signaling).

### Western blot analysis

A431, A549 or H1299 cells were seeded in 10-cm culture plates (1.2×10^6^ cells/plate) in DMEM 10% FBS, and then starved additional 24 h in serum-free medium. Cells, with and without AvidinOX conjugation (100 μg/mL), were then cultivated 18 h with MAbs or bMAbs at different concentrations in DMEM. In some experiments, antibodies were removed after initial 15 min contact. EGFR activation was performed by adding 100 ng/mL EGF (R&D) 30 min before cell lysis. At the end of culture, cells were washed twice with ice-cold PBS and then whole cell lysate was prepared by incubation, 10 min on ice, with 1× Lysis Buffer (Cell Signaling) supplemented with protease and phosphatase inhibitors. Cell lysates were subjected to sonication prior to centrifugation at 14,000 x g, for 10 min at 4°C, to remove cell debris. To extract the cytoplasmic/membrane protein fraction, the cells were washed twice with PBS and resuspended in low-salt buffer (20 mM Hepes, 1 mM EDTA, 1 mM EGTA) with 0.5% Nonidet P-40, 1 mM DTT and protease and phosphatase inhibitors and were allowed to swell on ice 20 min. The cell suspension was then transferred into a syringe and slowly passed 3 times through a 28-gauge needle, and subsequently subjected to sonication 10 sec on ice, followed by centrifugation 10 min at 4°C. The supernatant [cytosol/membrane fraction] was collected and stored at −80°C. The nuclear pellet was washed 3 times with cold low-salt buffer and resuspended in high-salt lysis buffer (20 mM Hepes, 1 mM EDTA, 1 mM EGTA, 420 mM NaCl, 20% glycerol) with DTT and protease and phosphatase inhibitors. Nuclear protein extraction was achieved by passing the solution through a syringe with a 28-gauge needle, and then incubating 30 min on ice before proceeding with the final sonication and centrifugation steps as above. The supernatant was snap-frozen into aliquots and stored at −80°C. Protein content was determined by Bradford method. Equal amounts of soluble proteins were separated on SDS-PAGE and then transferred to nitrocellulose membrane (Amersham Hybond-ECL; GE Healthcare). Membranes were blocked 3 h at room temperature with 5% non-fat dry milk in PBS 0.05% Tween-20 (PBS-T) before overnight incubation, at 4°C, with one of the following primary antibodies: pEGFR (#2236), EGFR (#4267), pAKT (#4058), AKT (#9272), pERK 1/2 (#9101) and ERK 1/2 (#9102) from Cell Signaling; pSTAT3 (#sc-81523) and STAT3 (#sc-7179) from Santa Cruz Biotechnology. Immunoblotting with anti-α-tubulin (#T5168; Sigma Aldrich) or anti-HDAC2 (#2540; Cell Signaling) antibodies was done to confirm equal protein loading for non-nuclear and nuclear protein extracts, respectively. After washings with PBS-T, membranes were incubated 1 h with the appropriate secondary HRP-conjugated anti-rabbit (1:3,000) or anti-mouse (1:2,000) IgG antibody (Sigma Aldrich and Amersham GE-Healthcare, respectively). Immunoreactive bands were visualized after enhanced chemiluminescence detection (Amersham ECL plus; GE-Healthcare) and analyzed by phosphoimaging (STORM, Molecular Dynamics) or by exposure to X-ray film (Amersham Hyperfilm ECL; GE-Healthcare).

### EGFR dimerization Analysis

Cross-linking experiments were essentially performed as described by Panosa *et al*. [49] Briefly, A549 and H1299 cells, with or without AvidinOX conjugation (100 μg/mL), were treated 15 min with bMabs and harvested in ice-cold lysis buffer [20 mM sodium phosphate pH 7.4, 150 mM NaCl, 1% Triton X-100, 5 mM EDTA, protease and phosphatase inhibitors], after 5 min or 18 h cultivation in serum-free medium. Aliquots of cell lysates were stimulated or not with 1 μg/mL EGF, 30 min at room temperature. Then receptor cross-linking was induced by incubating 2 min with 40 mM glutaraldehyde and reaction was stopped by addition of 0.2 M glycine (pH 9.0). Protein samples were finally assessed by Western Blot with the following primary antibodies: EGFR (#05-104) from Upstate, and ErbB2 (Neu A-2; #sc-393712) from Santa Cruz Biotechnology.

### Nuclear EGFR ELISA

AvidinOX-conjugated (100 μg/mL) A549 and H1299 cells were treated 15 min with Mabs or bMabs at different concentrations and then cultivated 18 h in fresh medium, with or without EGF (100 ng/mL) induction the last 30 min. Nuclear protein extracts were prepared as described above and EGFR content assessed by the PathScan Total EGF Receptor Sandwich ELISA kit (Cell Signaling), according to manufacturer's instructions.

### Animal studies

All studies were conducted in accordance with European Directive 86/609, Italian Legislation D.L. 116, Art. 6 1992 and ARRIVE guidelines [50].

### A549 orthotopic tumor model and efficacy studies

Metastatic lung cancer was established by injecting 6×10^6^ A549 or A549-luc-C8 (A549luc) cells into the tail vein of athymic nude or SCID/beige mice. After 1-3 weeks the mice were randomized in groups of 12 and treated by nose-only (Inexpose System, Scireq-EMKA Technologies) or whole body (AirFamily system, Pic indolor) aerosol with AvidinOX (6.5 mL of 3 mg/mL solution) followed, after 4 h, by nebulized PBS (antibody vehicle) or bCet. Treatments were repeated for 4-8 consecutive weeks and death events recorded. Control groups were i.v. administered 1 mg/mouse Cetuximab. Tumor bioluminescence imaging (BLI) was recorded at different time points by Xenogen IVIS Imaging System 200 (Perkin Elmer), 15 min after i.p. injection of luciferin (150 μg/mouse). Details on nose-only and whole body equipment and dose calculation in Supplementary Figure S5B,C.

### Tolerability and immunogenicity

Balb/c mice (6 mice/group) were treated by nose-only or whole body aerosol with AvidinOX (6.5 mL of 3 mg/mL solution), with or without subsequent 3.5 mL of 30 μg/mL murine biotinylated IgG or bCet nebulization, four hours later. Treatment was performed once a week for 6-8 consecutive weeks. One and 7 days after the last nebulization, mice were sacrificed and blood collected for mouse IgG and IgA anti-AvidinOX antibody (MAVA) titration. Lungs were explanted, bronchoalveolar lavage (BAL) collected and used for multi-cytokine detection (SearchLight multiplex immunoassay kit). Lung samples were fixed in formalin for histology evaluation. MAVA titration was performed by ELISA as previously described [17] on AvidinOX-coated plates using an AP-conjugated goat anti-mouse IgG or a HRP-conjugated rat anti-mouse IgA (Sigma Aldrich). The titer was expressed as log_2_ dilution giving OD=0.5. Confirmatory assay was performed in positive samples by adding an excess of free avidin. AvidinOX and bCet were administered to Cynomolgus monkey by intratracheal laryngoscope-guided nebulization. Three groups of two young females of 3.5-4.8 kg were treated, under general anaesthesia, with 3.0 mg/kg AvidinOX, 12.6 mg/animal bCet or AvidinOX followed by b-Cet at the same doses, 15 min apart. Nebulization was performed by AeroProbe (Trudell Medical International). The animals were continuously observed for behavioural changes (i.e. food consumption) and clinical signs. To evaluate the systemic exposure to AvidinOX or bCet, blood samples were collected before and at different time points after nebulization and sera tested by specific ELISA. Forty height hours after treatment animals were euthanized by sodium thiopental for body and organ weight, necropsy, lung histology and immunohistochemistry analyses. The study was conducted at Accelera (Milan, Italy). Immunohistochemistry was performed on paraffin embedded sections both for mice and cynomolgus studies. For avidin and Cetuximab staining, rabbit anti-avidin polyclonal antibody (Abnova) and rabbit anti-human IgG polyclonal antibody (Thermo Scientific) were used at 1:1,000 and 1:500 working dilutions, respectively, followed by the ImmPress anti-rabbit detection complex (Vector). Negative controls consisted in the omission of primary antibody and untreated animals.

### Statistical evaluations

Independent two-sample t-test was used for comparing experimental versus control groups, according to sample size and variance. Statistical analysis of multiple groups' comparison (more than three groups within a single experiment or two groups in repeated experiments) was performed using ANOVA. A significance threshold of p≤0.05 was assumed. In all figures, values were expressed as the mean ± standard deviation or standard error. Survival analyses were carried out by the Kaplan-Meier method and survival time differences were assessed using log-rank analysis. All analyses were accomplished using SigmaPlot software (Systat Software Inc., San Jose, CA, USA).

## SUPPLEMENTARY, TABLE AND FIGURE


