# Supplementary material for: Efficacy of aerosol therapy of lung cancer correlates with EGFR paralysis induced by AvidinOX-anchored biotinylated Cetuximab
Source: Oncotarget. 2014 Aug 31;5(19):9239–55. doi: 10.18632/oncotarget.2409 (PMC4253431; doi:10.18632/oncotarget.2409)
Supplement: Supplementary file 1 [file oncotarget-05-9239-s001.pdf]

Efficacy of aerosol therapy of lung cancer correlates with EGFR paralysis induced by AvidinOX-anchored biotinylated Cetuximab

Supplementaray Material

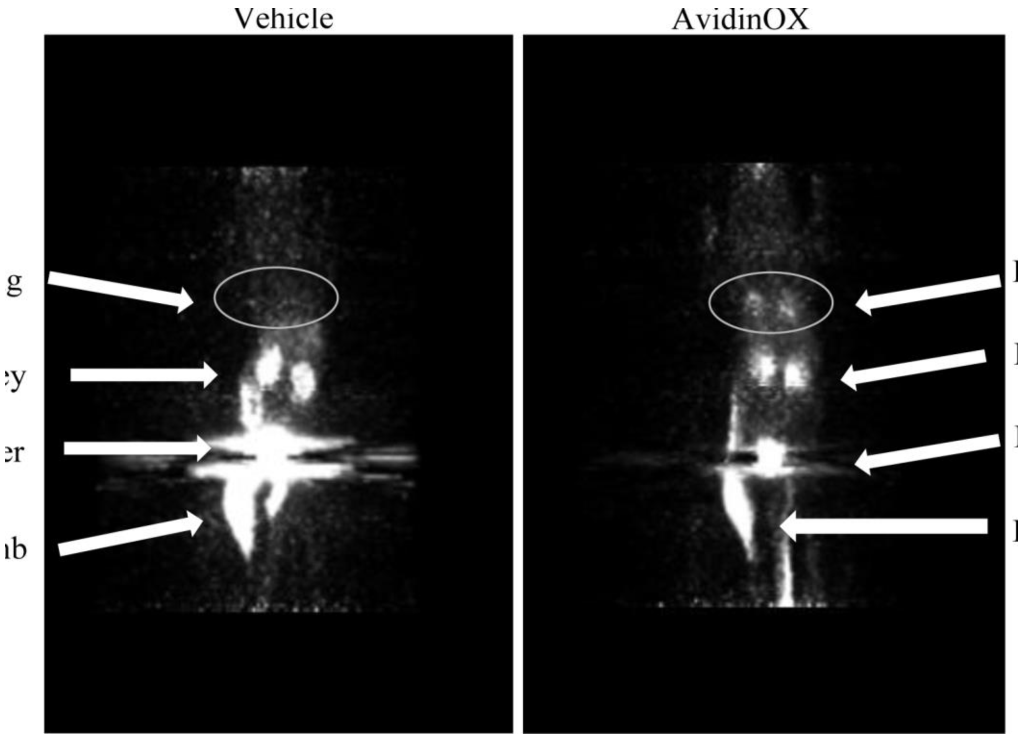

Supplementary Figure S1: Lung uptake of intravenous ^{64}Cu-ST2210 in mice after aerosol treatment with AvidinOX Groups of balb/c mice of 8 weeks (*n* = 3) were nebulized with 3.0 mg/mL AvidinOX solution or vehicle, 40 min by nose-only equipment. AvidinOX (15 µL, 3.0 mg/mL) was also injected in the hind limb muscle of all mice as an internal positive control. Each mouse received intravenous ^{64}Cu-ST2210 (7-8 MBq), 24 h after aerosol. Whole body µPET images were acquired by Explore Vista PET system (GE Healthcare), 16 h post injection. Mice were anesthetized during µPET scanning and ECG and vital signs were constantly monitored. The µPET scans were reconstructed using the ordered subset expectation maximization (OSEM) algorithm. Representative images indicate radioactivity uptake in AvidinOX-treated limbs and in lung of AvidinOX but not vehicle nebulized mice. The study was conducted at NSA, Ardea, Italy.

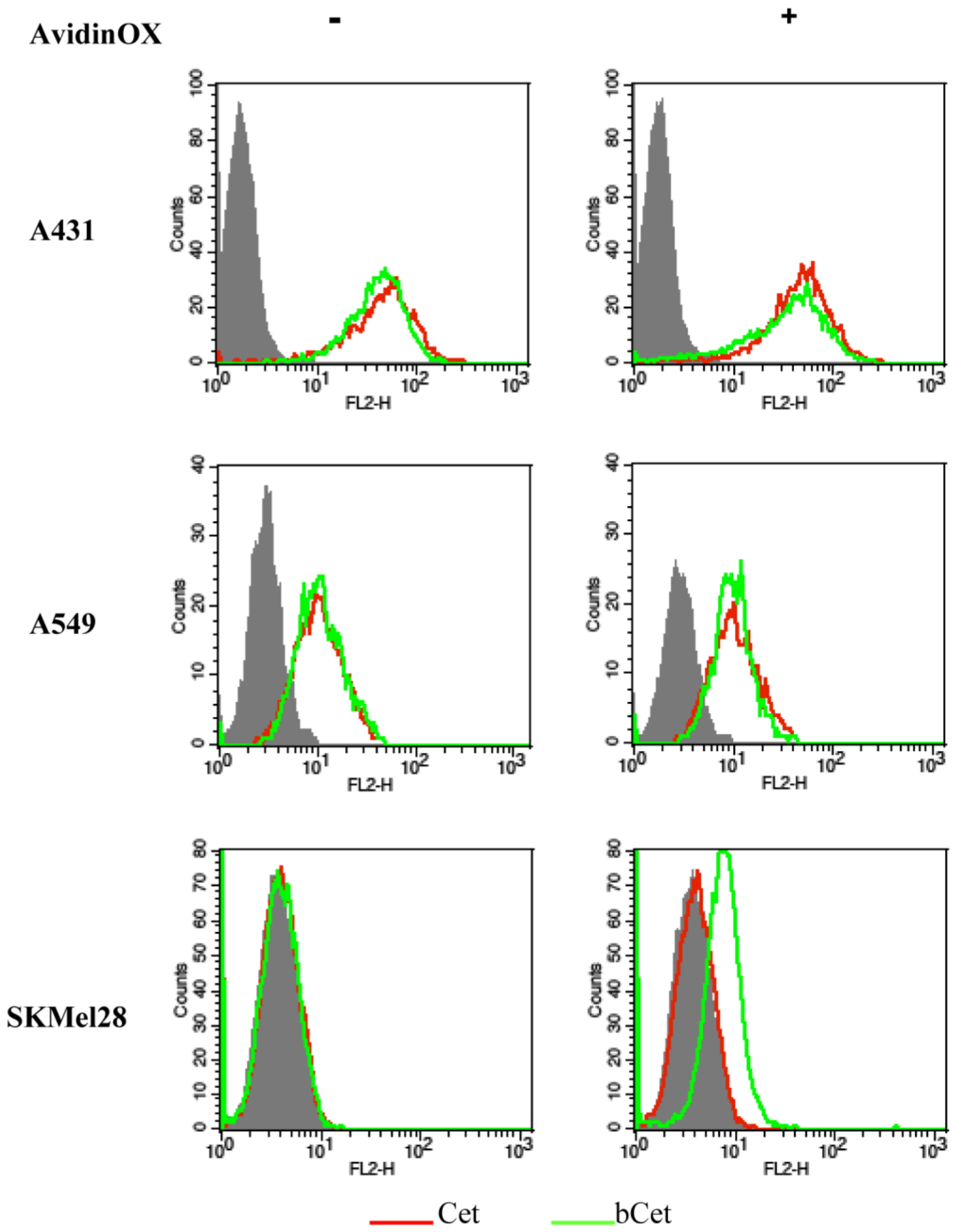

Supplementary Figure S2A: Specific binding of Cetuximab to tumor cells is not affected by AvidinOX conjugation Cytofluorimetry of indicated cells, without or with AvidinOX conjugation (100 µg/mL), incubated with 5 µg/mL Cet or bCet. Detection of antibody binding by phycoerythrin-conjugated mouse anti-human Ig (BD). Grey area, secondary antibody only.

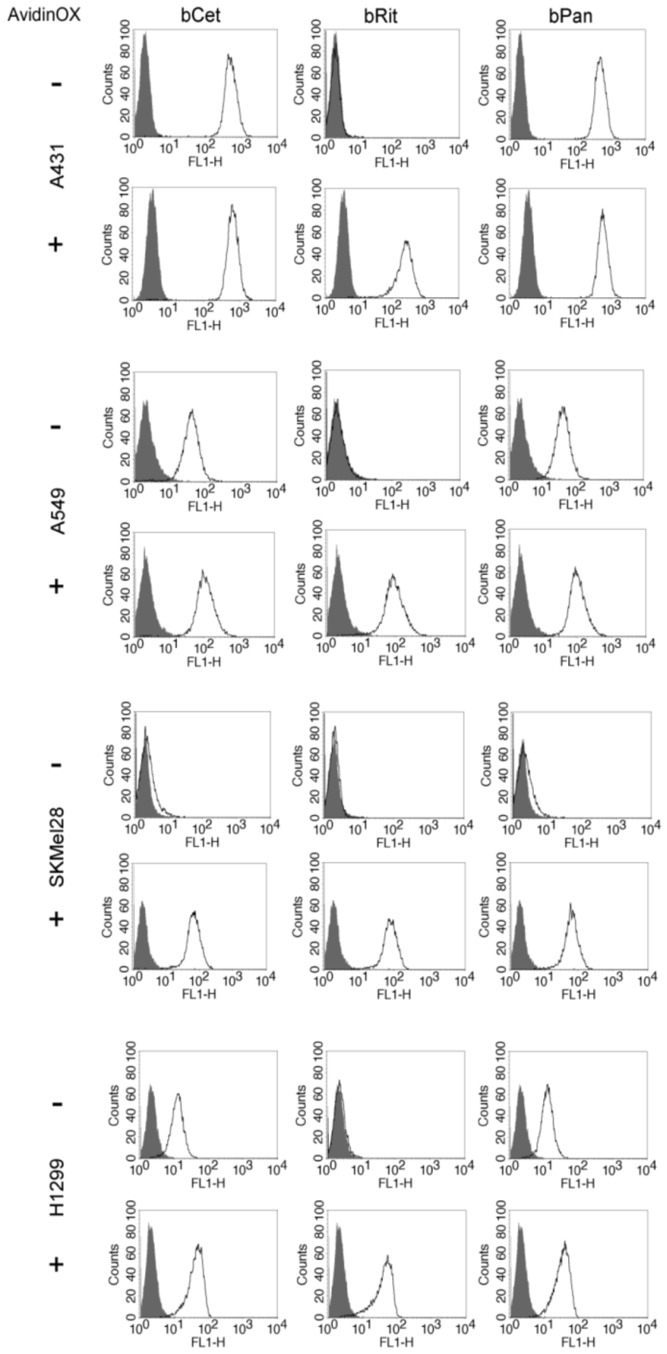

Supplementary Figure S2B: Binding of bCet, bPan and bRit to tumor cells with and without AvidinOX conjugation Cytofluorimetry of the indicated cells, without or with AvidinOX conjugation (100 µg/mL), incubated with 5 µg/mL CF488-labelled bCet, bRit or bPan. Grey area, cells without antibody.

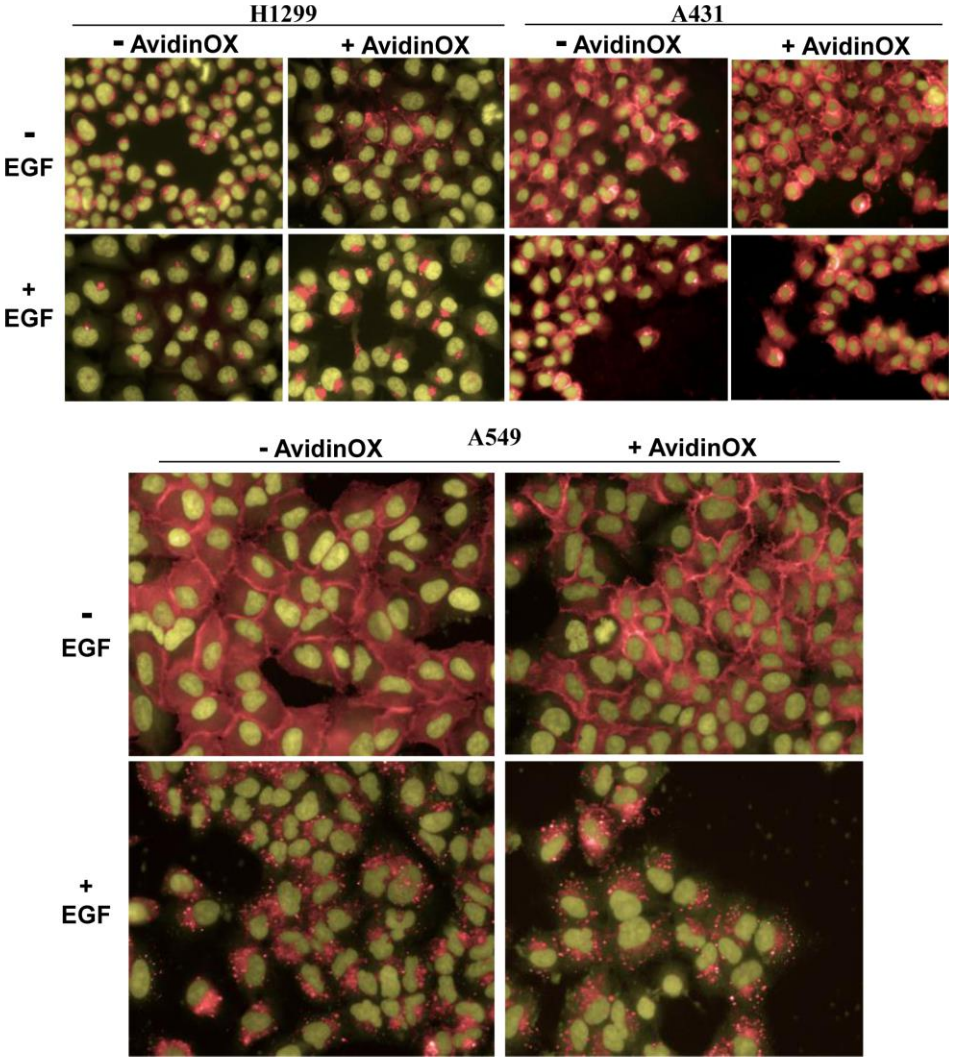

Supplementary Figure S3A: EGF-induced EGFR endocytosis in tumor cells is not affected by AvidinOX conjugation Cells, without or with AvidinOX conjugation (100 µg/mL), incubated 30 min with 20 nM EGF. After washing, the cells were fixed and stained for the detection of EGFR by using AF555-labeled anti- EGFR D38B1 antibody (red signal). Draq5-stained nuclei and cytoplasm in yellow. Fluorescence images were acquired by High Content Screening (HCS) Operetta. Each picture is representative of at least 5 fields of triplicate wells. 60X magnification.

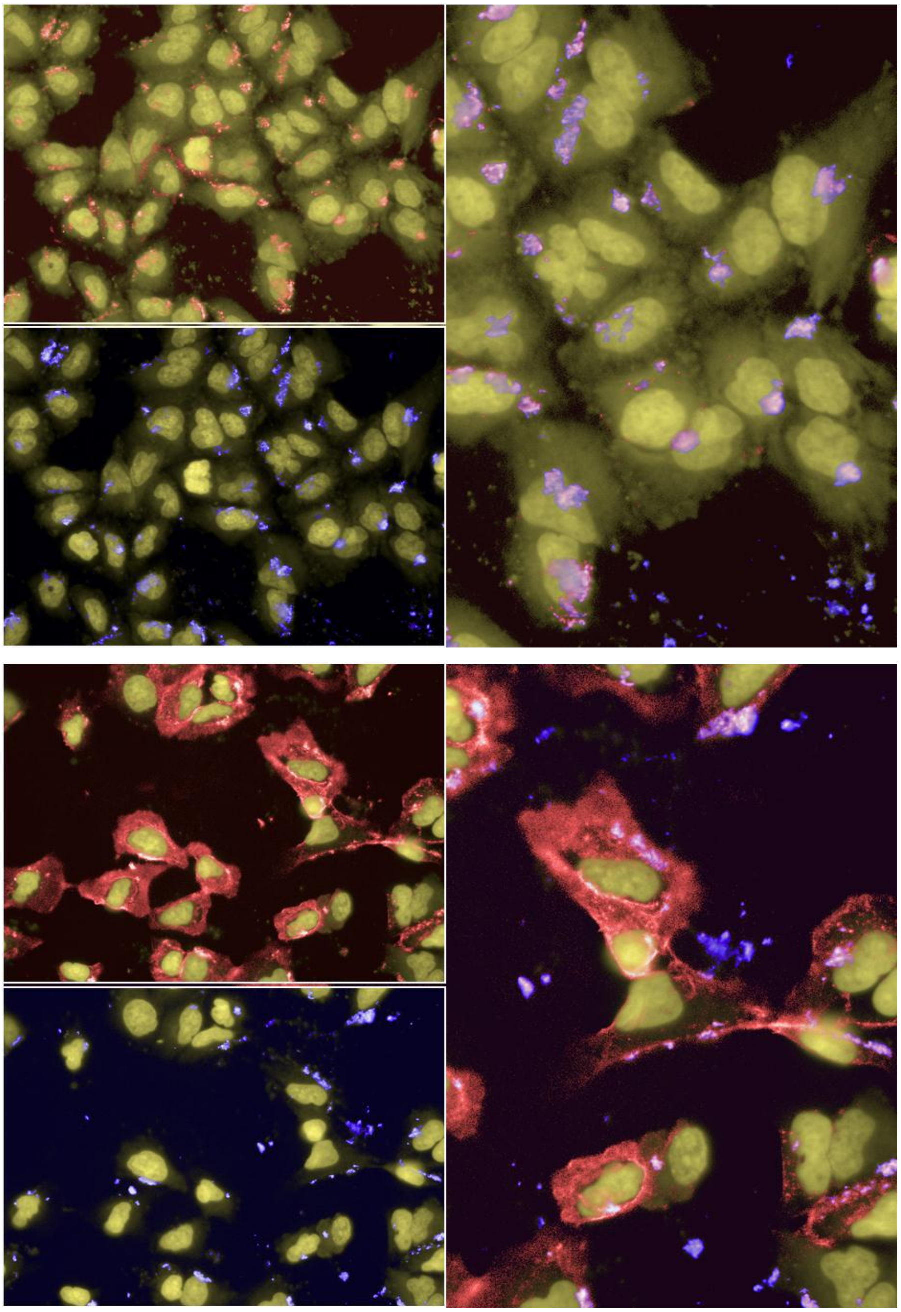

Supplementary Figure S3B: Co-localization of bCet and EGFR on the cell membrane of AvidinOX-conjugated A549 cells is associated to disappearance of intracellular EGFR AvidinOX-conjugated cells (100 µg/mL), incubated 2 h with 5 µg/mL CF488-labelled bCet or bRit (blue signal). After washing, cells were fixed and stained for the detection of EGFR by AF555-labeled anti-EGFR D38B1 antibody (red signal). Draq5-stained nuclei and cytoplasm in yellow. Violet signal in the merged images is the result of blue and red dye co-localization. Fluorescence analysis was performed by High Content

Screening (HCS) Operetta. Each picture is representative of at least 5 fields of triplicate wells. Magnification 60X.

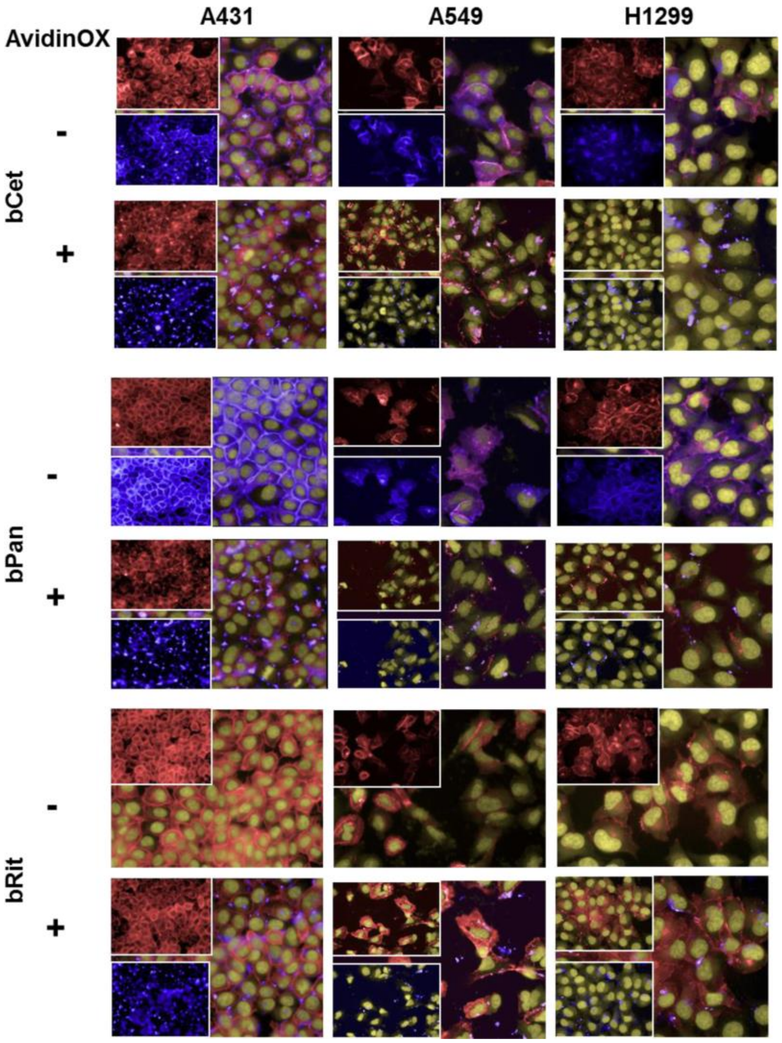

Supplementary Figure S3C: bCet, bPan but not bRit inhibit endocytosis and induce disappearance of intracellular EGFR in AvidinOX-conjugated cells. Cells, without or with AvidinOX conjugation (100 µg /mL), incubated 2 h with 5 µg/mL CF488- labelled bCet, bPan or bRit (blue signal). After washing, cells were fixed and stained for the detection of EGFR by AF555-labeled anti-EGFR D38Bl antibody (red signal). Draq5-stained nuclei and cytoplasm in yellow. Violet signal in the merged images is the result of blue and red dye co- localization. Fluorescence analysis was

performed by High Content Screening (HCS) Operetta. Each picture is representative of at least 5 fields of triplicate wells. Magnification 60X.

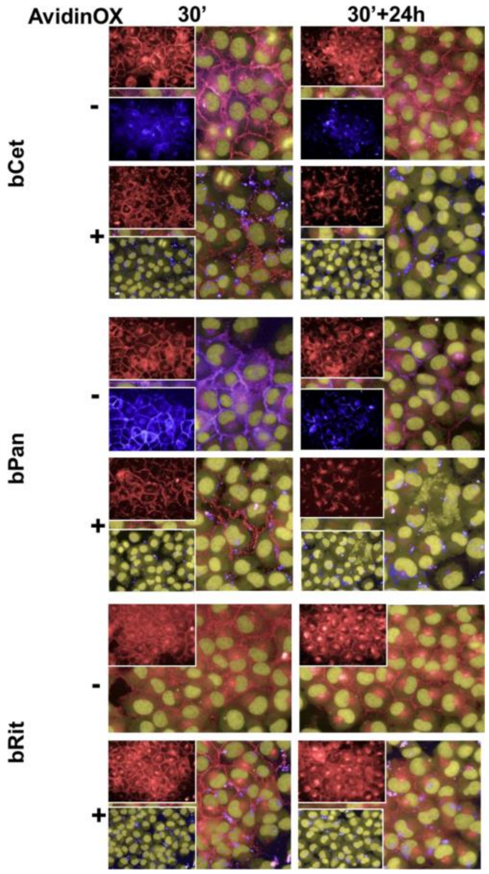

Supplementary Figure S3D: bCet, bPan but not bRit inhibit endocytosis and induce disappearance of intracellular EGFR in AvidinOX-conjugated H1299 cells Cells, without or with AvidinOX conjugation (100 µg/mL), incubated 30 min with 5µg/mL CF488- labelled bCet, bPan or bRit (blue signal). After washing, cells were fixed and stained for the detection of EGFR by AF555-labeled anti-EGFR D38B1 antibody (red signal), immediately or after 24 h cultivation. Draq5-stained nuclei and cytoplasm in yellow. Violet signal is the result of blue and red dye co-localization in the merged images. Fluorescence analysis was performed by HCS Operetta. Each picture is representative of at least 5 fields of triplicate wells. Magnification 60X.

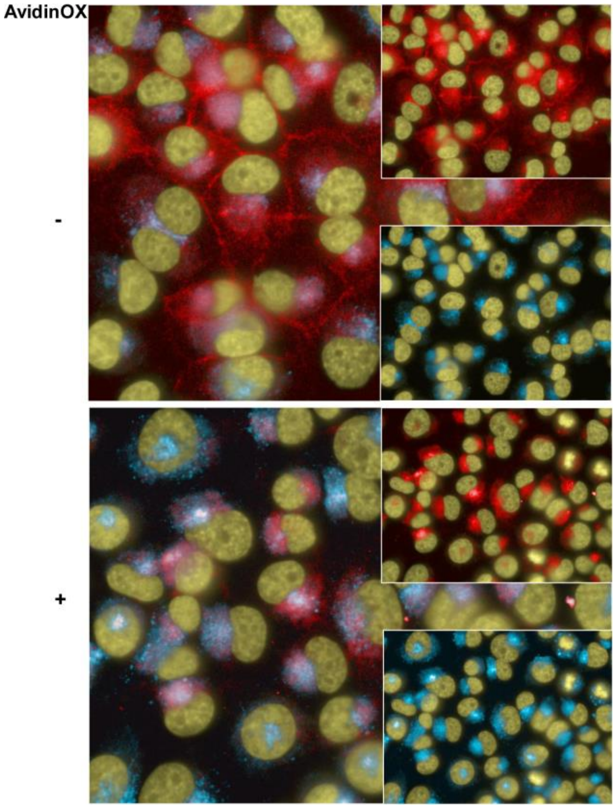

Supplementary Figure S3E: AvidinOX-anchored bCet induces EGFR and lysosome co-localization A549 cells, without or with AvidinOX conjugation (100 µg/mL), incubated 30 min with 1 µg/mL bCet. After washing, cells were fixed and stained for the detection of EGFR by AF555-labeled anti-EGFR D38B1 antibody (red signal), after 24 h cultivation. Lysosomes were stained by LysoTracker probe (light blue signal). Nuclei were stained by Hoechst (yellow signal). Violet signal is the result of blue and red dye co-localization in the merged images. Fluorescence analysis was performed by HCS Operetta. Each picture is representative of at least 5 fields of triplicates wells. Magnification 60X.

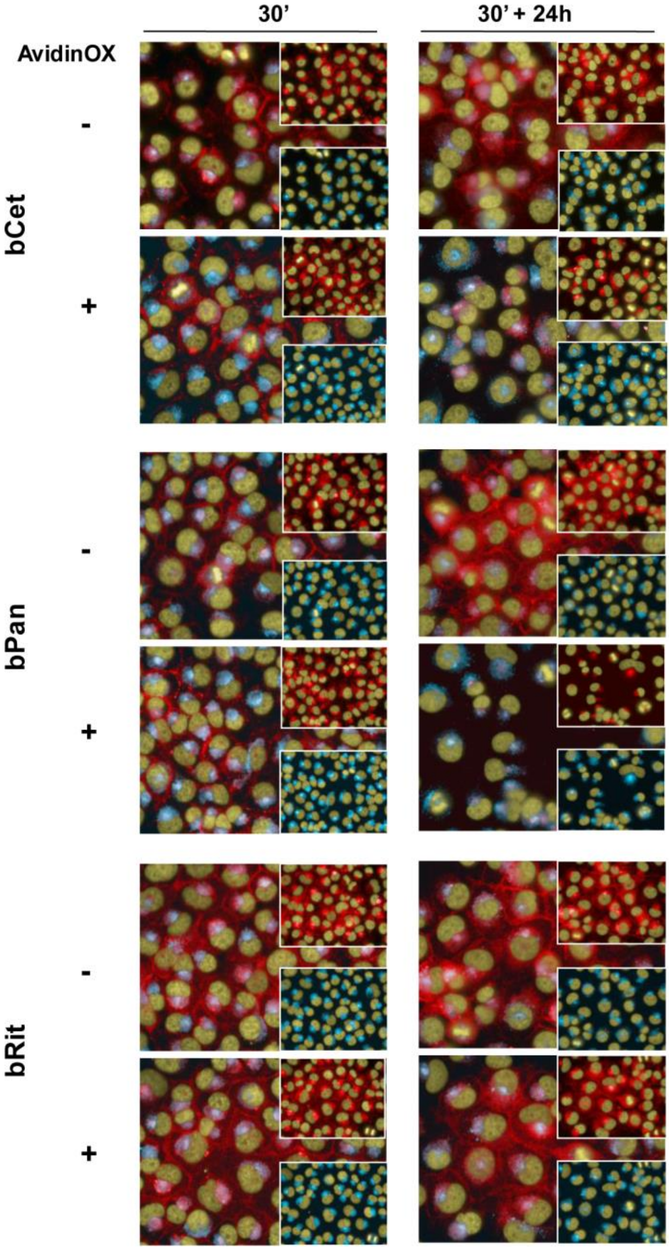

Supplementary Figure S3F: AvidinOX-anchored bCet, bPan but not bRit induce EGFR and lysosome co-localization A549 cells, without or with AvidinOX conjugation (100 μg/mL), incubated 30 min with 1 μg/mL bCet, bPan or bRit. After washing, cells were fixed and stained for the detection of EGFR by AF555-labeled anti-EGFR D38B1 antibody (red signal), immediately or after 24 h cultivation. Lysosomes were stained by LysoTracker probe (light blue signal). Nuclei were stained by Hoechst (yellow signal). Violet signal is the result of blue and red dye co-localization in the merged images.

Fluorescence analysis was performed by HCS Operetta. Each picture is representative of at least 5 fields of triplicates wells. Magnification 60X.

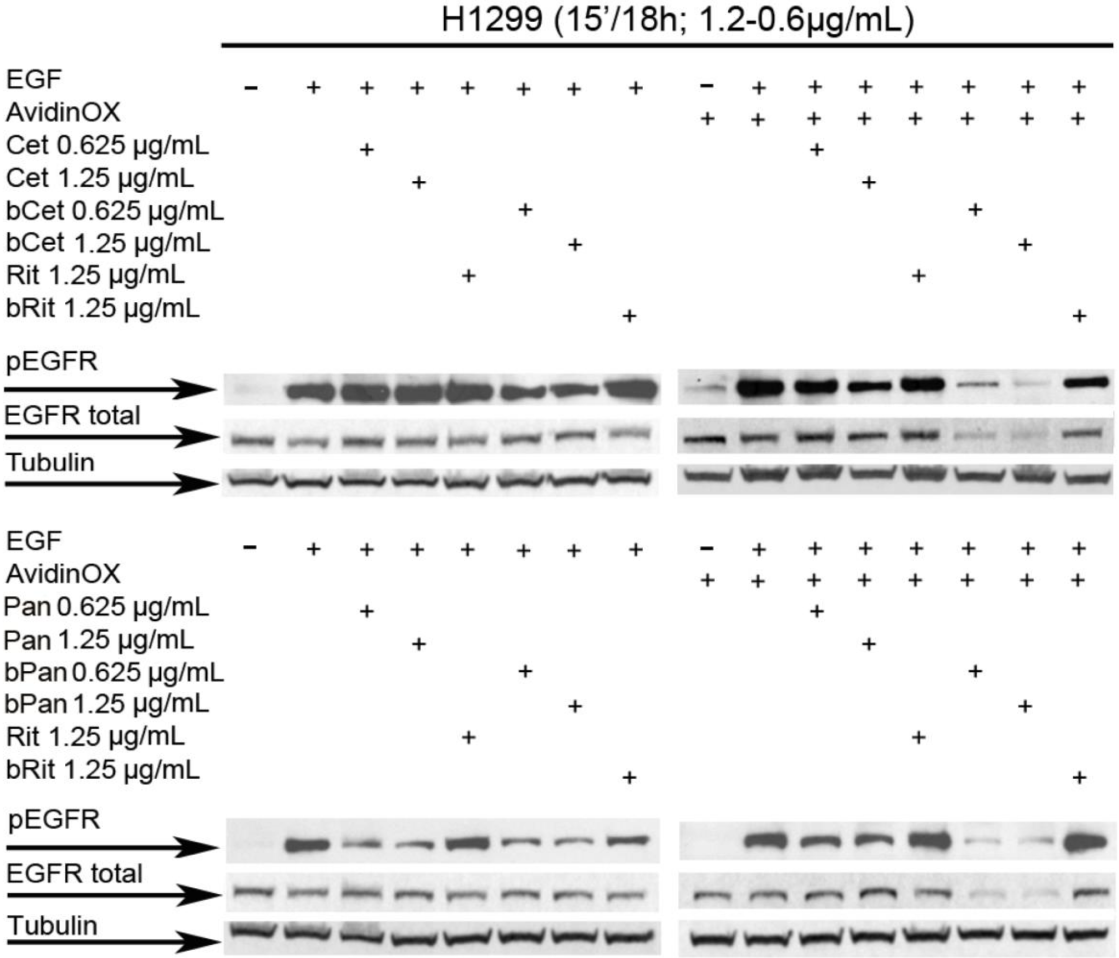

Supplementary Figure S4: AvidinOX-anchored bCet and bPan but not bRit inhibit EGFR phosphorylation and induce EGFR degradation in H1299 cells Cells, without or with AvidinOX conjugation (100 μg/mL), were serum-starved 24 h and then cultivated at indicated conditions. EGF (100 ng/mL) was added 30 min before cell lysis. Whole cell lysates were subjected to Western blot analysis with anti-pEGFR (Tyr1068), anti-EGFR and anti- tubulin antibodies.

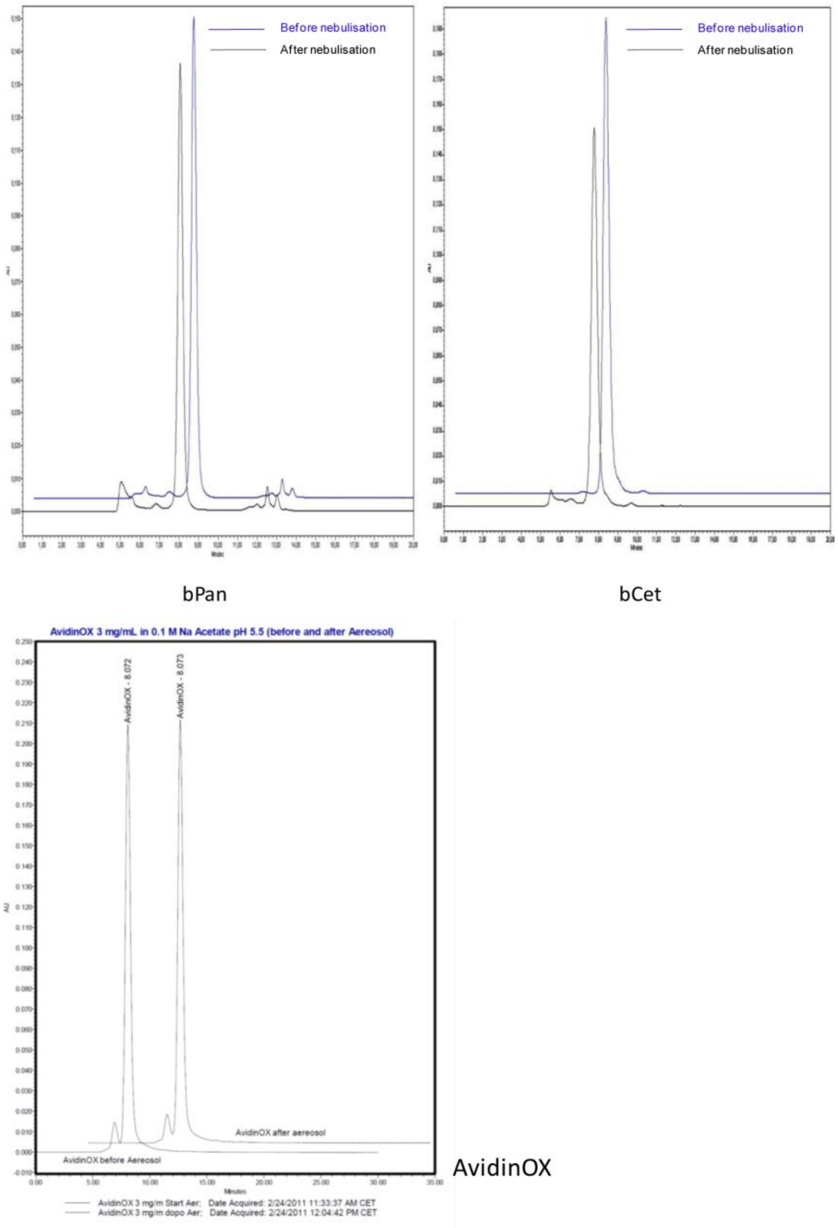

Supplementary Figure S5A: Size exclusion chromatography of bCet, bPan and AvidinOX confirmed integrity of nebulized proteins AvidinOX (3 mg/mL), bCet and bPan (1 mg/mL) solutions were nebulized by using Nose-Only Inexpose System aeroneb. Nebulized material was collected by conveying the mist in a falcon tube and condensed solutions were analyzed by SEC-HPLC in

comparison to pre-nebulized samples. AvidinOX was analyzed by BIOSEP S3000 and antibodies by TSKgel G3000 SWXL column, respectively.

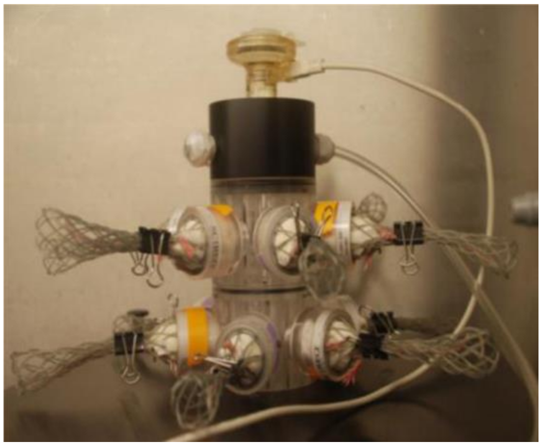

Supplementary Figure S5B: Nose-only aerosol equipment and dose calculation A549 transplanted mice were treated by aerosol with Nose-Only Inexpose System, Scireq-EMKA Technologies Aeroneb producing particles of 1-5 µm Delivered dose (DD) was calculated according to Alexander at al[1]

$$DD\ (mg/kg) = \frac{(C * RMV * D * IF)}{BW}$$

**AvidinOX 20 mg (3.0 mg/mL solution)**	**bCetuximab 0.1 mg (30 µg/mL)**
C (Concentration of AvidinOX in air): 20 mg nebulized in 40 minutes by air flow rate 1L/minute= 0.5 mg/L<br><br>RMV (Respiratory Minute Volume): 0.045 L/minute, measured in a preliminary study by plethysmography on restrained mice<br><br>D (duration of exposure) = 40 minutes<br><br>IF(for used apparatus)= 0.4<br><br>BW (Body Weight) = 0.02 kg<br><br>$DD = \frac{(0.5*0.045*40*0.4)}{0.02}$= 18 mg/kg=<u>360µg/mouse</u><br><br>Lung delivered dose (LDD) estimated 20% of DD= **72 µg/mouse**	C (Concentration of bCet in air): 0.1 mg nebulized in 20 minutes by air flow rate 1L/minute= 0.005 mg/L<br><br>RMV (Respiratory Minute Volume): 0.045 L/minute, measured in a preliminary study by plethysmography on restrained mice<br><br>D (duration of exposure) = 20 minutes<br><br>IF (estimation for used apparatus) = 0.4<br><br>BW (Body Weight) = 0.02 kg<br><br>$DD = \frac{(0.005*0.045*20*0.4)}{0.02}$= 0.09 mg/kg=<u>1.8µg/mouse</u><br><br>Lung delivered dose (LDD) estimated 20% of DD= **0.36 µg/mouse**<br><br>LDD bCet 1 mg (100 µg/mL) : 3.6 µg/mouse<br><br>LDD bCet 10 mg (3 mg/mL): 36 µg/mouse

1. Alexander DJ *et al*. Association of Inhalation Toxicologists (AIT) working party recommendation for standard delivered dose calculation and expression in non-clinical aerosol inhalation toxicology studies with pharmaceuticals. *Inhal. Toxicol.* **20**, 1179-1189 (2008).

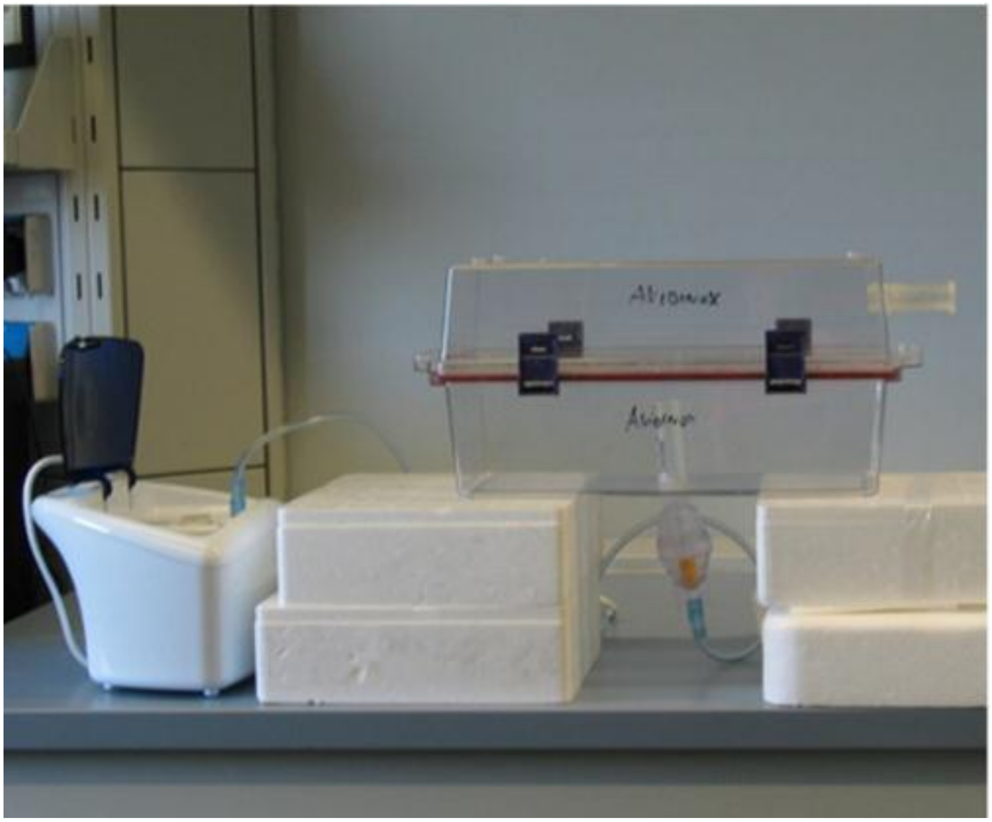

Supplementary Figure S5C: Whole body aerosol therapy of A549luc lung cancer with AvidinOX and bCet by using a household equipment Household equipment used for whole body nebulization. Lung Delivered Dose (LDD) estimated as in Supplementary **Fig. S5B** with the following parameters: Air Flow Rate 5 L/min; RMV 0.07 L/min. Nebulization volume 3.5 mL.

LDD AvidinOX 3 mg/mL (20 mg): 22.4 µg/mouse
LDD bCet 100 µg/mL (350 µg): 0.4 µg/mouse
LDD bCet 30 µg/mL (105 µg): 0.12 µg/mouse
LDD bCet 10 µg/mL (35 µg): 0.04 µg/mouse
LDD bCet 3 µg/mL (10 µg): 0.012 µg/mouse

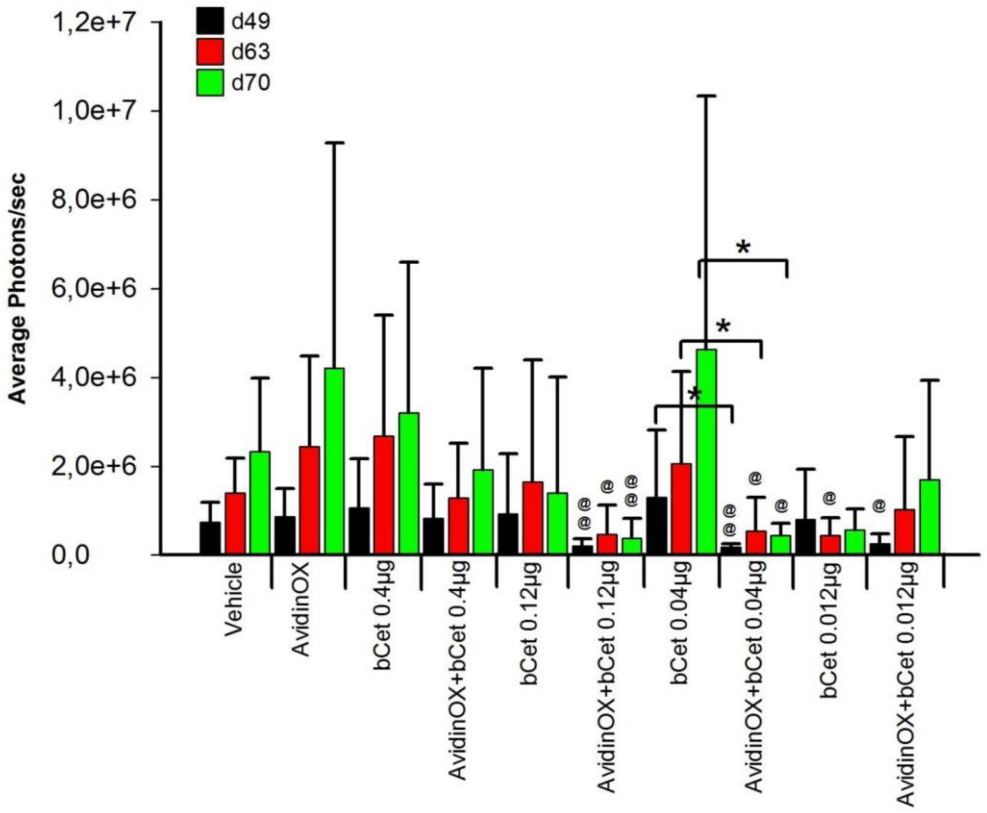

Supplementary Figure S5D: Aerosol treatment with AvidinOX and bCet is effective against A549 metastatic lung cancer Average photons of SCID/bg mice, i.v. injected with A549luc cells. Treatment started one week after tumor challenge. Measurements were performed after 6th and 8th treatment (d49 and 63, respectively) and one week after the last treatment (d70). Data are mean photons ± s.d. Student's *t*-test versus bCet:

*, *p*< 0.05. Student's *t*-test versus AvidinOX: @, *p*< 0.05; @@, p< 0.01.

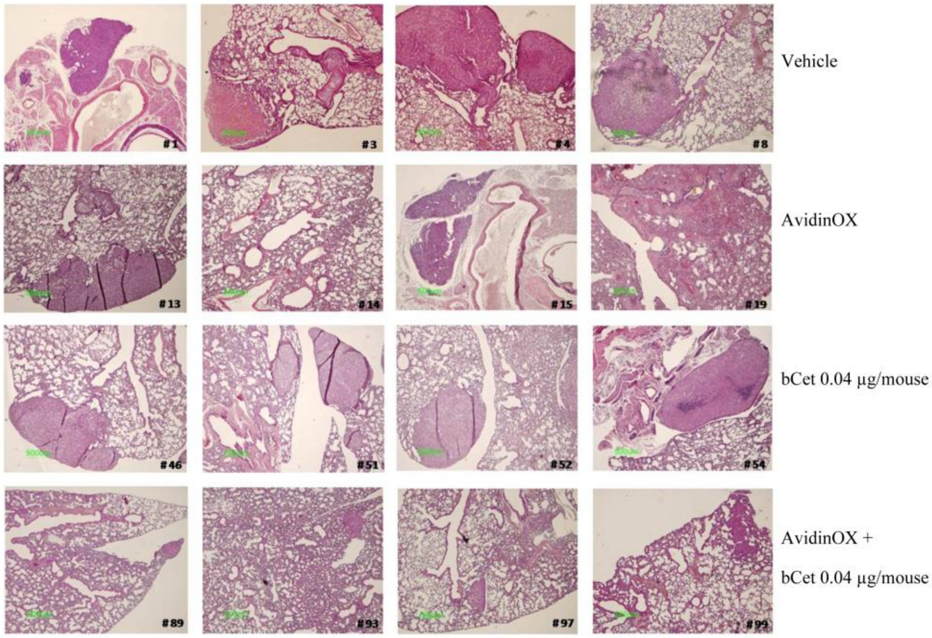

Supplementary Figure S5E: Lung histology of mice with A549luc lung cancer shows dramatic reduction of tumor burden after aerosol treatment with AvidinOX and bCet Representative pictures of H/E staining of serial sections of lungs from 4 mice of study in Figure 6H-L, sacrificed one week after last treatment (d70).

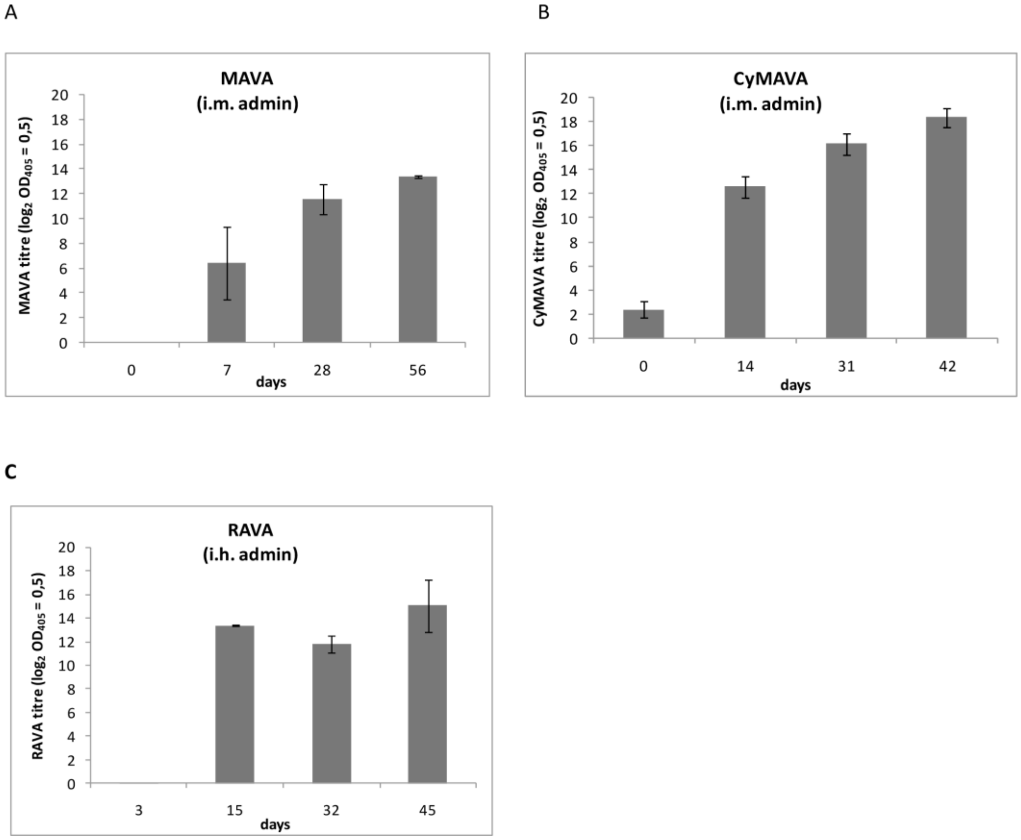

Supplementary Figure S5F: Anti AvidinOX antibody titers in different species **A,** Balb/c mice (n=9) treated once i.m. at day 0 with 50 µg AvidinOX. Three mice were sacrificed at indicated time points for MAVA titration. **B,** Cynomolgus monkeys treated i.m. with 22 mg AvidinOX at day 0 and 30 (n=8, 4 Male + 4 Female). Blood samples were collected at indicated time points for CyMAVA titration. **C**, Sprague dawley rats (n=36) treated intra hepatic (i.h.) with 450 µg AvidinOX at day 0 and 30. Twelve (6 Male + 6 Female) were sacrificed at indicated time points for RAVA titration. Titers are expressed as Log_2 serum dilution giving OD_{405}= 0.5. Error bars: mean ± s.d.

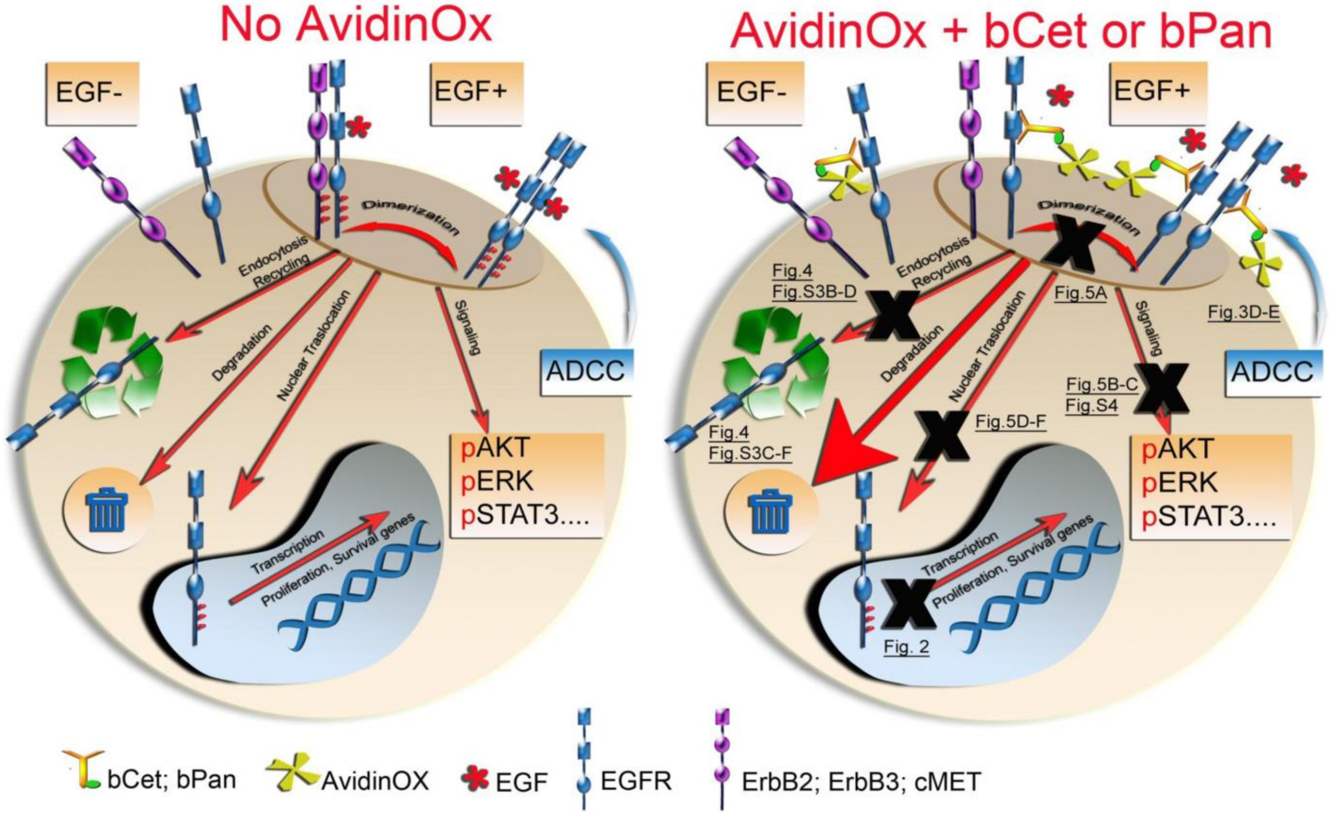

Supplementary Figure S6: Schematic representation of AvidinOX-dependent anti-EGFR antagonism by bCet and bPan

Supplementary Materials and Methods

Cell lines. A431, H1299 and SKMel28 were from ATCC, A549 and A549-luc-C8 were from DSMZ and Caliper, respectively. A431 and A549 were cultivated in DMEM 10% FBS, SKMel28 in the same medium with the addition of non- essential amino acids and H1299 and A549-luc-C8 in RPMI-1640 medium 10% FBS.

AvidinOX and biotinylated antibodies. AvidinOX® (registered brand of Sigma Tau) was prepared by Areta International as a lyophilized form, according to previously described methods (1). After reconstitution with water for injection, the protein was at 3.0 mg/mL, in acetate buffer pH 5.2 with mannitol and NaCl. For biotinylation of Cetuximab (Erbitux®; Merck Serono), Panitumumab (Vectibix®; Amgen) and Rituximab (MabThera®; Roche), 100 mg of antibody were subjected to buffer exchange by ultrafiltration on Amicon Ultra 30K (Millipore) and brought to a concentration of about 10 mg/mL in PBS. Activated 2X-AHbiotin-*N*-Hydroxysuccinimide ester (ST3297, Sigma-Tau) was added at 1:10 Mab:biotin molar ratio. The reaction mixture was incubated 2 h at room temperature, under mild shaking, loaded on SEC disposable PD-10 column (G-25 Amersham-Pharmacia) and eluted with PBS. Biotinylated antibody (bMab) fractions were pooled based on OD_{280} values and finally filter sterilized. The biotinylation level was assessed by HABA test (2) and purity and integrity confirmed by SEC-HPLC on TSKgel G3000 SWXL column (Tosoh Bioscience). Immunoreactivity was tested by antigen-specific ELISA. Briefly, Immuno MAXISORP 96-well plates (Nunc) were coated overnight at 4°C with 50 ng/well of recombinant human EGF-R/Erb1 Fc chimera (R&D). Plates were washed with PBS 0.1% Tween-20 (PBS-T), blocked with PBS-T 1% BSA for 2 h, and incubated with serial dilutions of antibodies, 1 h at room temperature. After washings, anti-human K light chain horseradish peroxidase (HRP)-conjugated antibody (Sigma Aldrich), diluted 1:1,000 in

blocking solution, was added 1 h at room temperature. After three washings, 200 µL/well TMB substrate (Sigma Aldrich) were added and plates incubated 30 min at 37°C. The reaction was blocked by adding 100 µL/well of 0.5M H_2SO_4 solution, and optical density at 450 nm measured by ELISA spectrophotometer. Endotoxins were tested by LAL test (Endosafe PTS/MCS Cartridges).

AvidinOX conjugation to cells. For AvidinOX conjugation, pellets of $5x10^5$ cells or adherent cells (96- or 24-well plates) were washed with PBS and incubated 1 h at 4°C with 100 µL of 10 or 100 µg/mL AvidinOX in PBS. Cells were then washed with DMEM and used for further *in vitro* experiments.

Antibody binding. Pellets of A431, A549, H1299 or SKMel28 cells, with or without AvidinOX-conjugation, were incubated 1 h at 4°C with Mabs or bMabs. After washings, cells were incubated with mouse anti-human PE-conjugated Ig (BD). Otherwise, Mabs and bMabs were directly labeled with CF488 dye according to manufacturer's instruction (Biotium). Cytofluorimetry was performed with FACScalibur (BD). Binding of CF488-labelled Mabs and bMabs was also assessed on 96-well plates ($2.5x10^5$ cells/well) after 1 h incubation at 4°C. After washings, fluorescence was evaluated by VICTOR2 plate reader (Perkin Elmer).

Proliferation assay. A431 ($3x10^3$), A549 ($1x10^4$), H1299 ($1x10^4$) and SKMel28 ($2x10^3$) cells/well (with and without AvidinOX conjugation), were seeded 24 h in 96 well-microtiter plates in complete DMEM medium. After washing, 200 µL Mabs or bMabs were added at different concentrations, in DMEM 1% FBS. The cells were maintained in the presence of antibodies for the entire duration of the culture, or washed after initial 15 min contact. Cell viability was assessed by the CellTiter-Glo

Luminescent Cell Viability Assay (Promega), XTT Cell Proliferation Kit II (Roche) or Cell Proliferation ELISA BrdU kit (Roche), according to manufacturer's instruction.

Clonogenic assay. Cells, with or without AvidinOX conjugation, were seeded at different density into 6 or 24 well plates in DMEM 1% FBS. Cet or bCet were added after 2 h. After 14 days, the cultures were washed twice with PBS, fixed with 70% ethanol and then stained with 0.05% crystal violet in PBS. Colonies were counted and then eluted with 30% acetic acid. Optical density at 595 nm recorded.

Apoptosis. Pellets of A431, A549 and SKMel28 cells ($5x10^5$ cells/mL), with and without AvidinOX conjugation, were cultivated with Mabs or bMabs in DMEM 10% FBS, 4 or 18 h. Cells were then washed with DMEM and stained with FITC-Annexin V apoptosis detection kit I (BD). All analyses were performed on FACScalibur cytometer using DIVA software package (BD).

Antibody-Dependent Cellular Cytotoxicity (ADCC). Effectors: Primary polyclonal NK cell cultures were obtained by 10-13 day co-culture of human PBMC with irradiated (30 Gy) Epstein-Barr virus-transformed B-cells (RPMI-8866) at 4:1 ratio, in RPMI-1640 medium 10% FBS, 2 mM glutamine, as previously described (3). Cultures were typically > 85% NK ($CD56^+CD16^+CD3^-$), as assessed by flow cytometry. Targets: A549 cells (with and without AvidinOX-conjugation) were labelled with ^{51}Cr (Perkin Elmer) (3.7MBq/1 x 10^6 cells) 75 min at 37°C; labelled cells were incubated with Mabs or bMabs at the indicated concentrations 20 min at room temperature, washed and plated ($5x10^3$/well) together with serial dilutions of NK effectors in U-bottomed 96-well plates in RPMI-1640 medium 10% FBS, 2 mM glutamine and 10 mM Hepes, 4 h at 37°.

Twenty-five µL of supernatant were transferred to 96-well Lumaplate (Perkin Elmer) and released radioactivity measured by β-counter (TopCount microplate scintillation counter, Perkin Elmer). Percent specific lysis was calculated according to the formula: % specific lysis = (experimental release cpm - spontaneous release cpm)/(maximum release cpm - spontaneous release cpm) x 100. Spontaneous release never exceeded 8% of total release.

Animal studies. All studies were conducted in accordance with European Directive 86/609, Italian Legislation D.L. 116, Art. 6 1992 and ARRIVE guidelines.

Lung distribution of inhaled AvidinOX and biotin uptake. Balb/c mice were exposed to vehicle (100 mM sodium acetate, pH 5.0) or 3.0 mg/mL AvidinOX solution by using the Nose-Only Inexpose System, Scireq-EMKA Technologies. For PET imaging, biotinDOTA (ST2210, Sigma-Tau) was radiolabeled at Nuclear Specialists Association (NSA), with ^{64}Cuprum using the automated synthesizer module TADDEO. Mice were exposed to AvidinOX or vehicle by aerosol as described above and intravenously injected, 24 h later, with 7-8 MBq ^{64}Cu-ST2210. Intramuscular injection of 45 µg of AvidinOX in one hind limb was performed in all mice as an internal positive control. Details of PET imaging in Supplementary Figure S2. For quantitative evaluation of the biotin uptake in lung and non-target organs, 10 MBq of ^{111}In-ST2210 were intravenously injected in groups of balb/c mice exposed to AvidinOX or vehicle by aerosol, 24 h earlier. The mice were sacrificed by asphyxia 2 or 24 h thereafter and lung, blood, spleen, liver, stomach, brain and kidney samples collected, weighed and counted by gamma counter (Cobra, Perkin Elmer). Data were expressed as % of the injected dose/gram of tissue (% ID/g). For immunohistochemistry, animals were exposed to nebulised AvidinOX and lungs were removed, fixed in formalin and paraffin embedded. Serial sections were processed and incubated with HRP-conjugated rabbit anti-avidin antibody (GeneTex) and then with DAB substrate.

1. Verdoliva A, Bellofiore P, Rivieccio V, Catello S, Colombo M, Albertoni C, Rosi A, Leoni B, Anastasi AM, De Santis R. Biochemical and Biological Characterization of a New Oxidized Avidin With Enhanced Tissue Binding Properties. *J Biol Chem* 2010;285:9090-9099.
2. Green NM. A spectrophotometric assay for avidin and biotin based on binding of dyes by avidin. *Biochem J* 1965;94:23C-24C.
3. Palmieri G, Serra A, De MR, Gismondi A, Milella M, Piccoli M, Frati L, Santoni A. Cross-Linking of Alpha 4 Beta 1 and Alpha 5 Beta 1 Fibronectin Receptors Enhances Natural Killer Cell Cytotoxic Activity. J Immunol 1995;155:5314-5322.

Supplementary Table S1A: Biodistribution of intravenous ^{111}In-ST2210 in AvidinOX or vehicle nebulized mice Balb/c mice of 5 weeks were put on biotin-free diet 4 days before exposure to vehicle or AvidinOX. Nebulization was performed by using the nose only equipment InExpose (EMKA Technologies). Groups of mice (n = 12) were exposed to 10, 6.7, 3.4 or 1.7 mL AvidinOX solution (3.0 mg/mL) and nebulization lasted 60, 40, 15 and 7 min, respectively. The mice of the vehicle group were treated for 60 min. Twenty four h after aerosol the mice were intravenously injected with 1 µg ^{111}In-ST2210 ($3x10^6$ MBq) and 2 h later they were sacrificed by asphyxia and lungs and non-target organs weighed and counted by gamma counter. Data are expressed as % of the injected dose/g of tissue (%ID/g). Student's t test: ***, p<0.001 versus vehicle; Student-Newman-Keuls: @, p<0.001 versus 15 and 7 minutes.

**Nebulized product**	**Exposure (min)**	**Blood %ID/g**	**Spleen %ID/g**	**Kidney %ID/g**	**Liver %ID/g**	**Lung %ID/g**
**Vehicle**	60	0.035±0.034	0.087±0.017	1.855±0.218	0.169±0.022	0.068±0.011
**AvidinOX**	60	0.027±0.019	0.078±0.015	1.785±0.236	0.160±0.022	1.673±0.119*** @
**AvidinOX**	40	0.623±0.826	0.082±0.014	1.620±0.438	0.136±0.029	1.933±0.403*** @
**AvidinOX**	15	0.314±0.206	0.081±0.013	1.500±0.249	0.347±0.570	0.836±0.303***
**AvidinOX**	7	0.862±0.848	0.093±0.023	1.611±0.264	0.300±0.438	1.154±0.547***

Supplementary Table S1B: Specifications of biotinylated Cetuximab, Panitumumab and Rituximab[#]

Test	Method	Expressed as	Specifications
**Biotinylation level**	HABA	Biotin moles/IgG mole	1 ± 0.3
**Purity**	SEC-HPLC	% peak area	>95
**Endotoxin**	LAL	EU/mL	<0.3
**Immunoreactivity**	ELISA	% versus not biotinylated	± 30

[#]Biotinylated Rituximab non tested for immunoreactivity

Supplementary Table S1C: Tumor cell line characteristics

Cell line	EGFR expression	KRAS	NRAS	Other activated pathways	Ref
**A431**<br>**Epidermoid carcinoma**	+++	WT	WT	cMet	[1-3]
**A549**<br>**Lung adenocarcinoma**	+	Mut<br>G12S	WT	JAK2<br>NF-KB	[2-5]
**H1299**<br>**Lung carcinoma**	++	WT	Mut<br>Q61K	MAPK<br>PI3K	[5-7]
**SKMel28**<br>**Melanoma**	-	WT	WT	B-RAFmt<br>V600E	[8, 9]

1. Jo, M. et al. Cross-talk between epidermal growth factor receptor and c-Met signal pathways in transformed cells. *J. Biol. Chem.* **275**, 8806-8811 (2000).

2. Kurai, J. et al. Antibody-dependent cellular cytotoxicity mediated by cetuximab against lung cancer cell lines.
Clin. Cancer Res. **13**, 1552-1561 (2007).

3. Schlaeth, M. et al. Fc-engineered EGF-R antibodies mediate improved antibody-dependent cellular cytotoxicity (ADCC) against KRAS-mutated tumor cells. *Cancer Sci.* **101**, 1080-1088 (2010).

4. Mijatovic, T. et al. The cardenolide UNBS1450 is able to deactivate nuclear factor kappaB-mediated cytoprotective effects in human non-small cell lung cancer cells. *Mol. Cancer Ther.* **5**, 391-399 (2006).

5. Yauch, R. L. et al. Epithelial versus mesenchymal phenotype determines in vitro sensitivity and predicts clinical activity of erlotinib in lung cancer patients. *Clin. Cancer Res*. **11**, 8686-8698 (2005).

6. Pallier, K. et al. TWIST1 a new determinant of epithelial to mesenchymal transition in EGFR mutated lung adenocarcinoma. *PLoS One* **7**, e29954 (2012).

7. Sunaga, N. et al. Knockdown of oncogenic KRAS in non-small cell lung cancers suppresses tumor growth and sensitizes tumor cells to targeted therapy. *Mol. Cancer Ther.* **10**, 336-346 (2011).

8. Hao, H. et al. Context-dependent roles of mutant B-Raf signaling in melanoma and colorectal carcinoma cell growth. *Mol. Cancer Ther*. **6**, 2220-2229 (2007).

9. Solit, D. B. et al. BRAF mutation predicts sensitivity to MEK inhibition. *Nature* **439**, 358-362 (2006).
